# Correction: A kinesin Klp10A mediates cell cycle-dependent shuttling of Piwi between nucleus and nuage

**DOI:** 10.1371/journal.pgen.1009147

**Published:** 2020-10-21

**Authors:** The PLOS Genetics Staff

The links to S4, S5, S6, S7, S8 and S11 Figs are not functioning correctly. Please view the figures here. The publisher apologizes for the error.

## Supporting information

S4 Fig Characterization of piRNA density across TE transcripts. A-B) Density of sequenced piRNAs (blue: sense; red: antisense) across *FB-element and BARI1*.
(PDF)

S5 Fig Ping-pong pathway activity remain unaltered after klp10A depletion. A) Histogram showing the distribution of antisense and sense piRNA pairs of piRNAs mapping to transposons. B) The box-plots show the distribution of ping-pong ratios of each transposon. Each box-plot is a different biological replicate. The Ping-pong ratio of each transposon was calculated by taking the sum of piRNA reads in which sense piRNAs with a 10 nt A and antisense piRNAs with a 1nt U showing 10 nucleotide complementarity from the 5’ end and dividing it with the total number of piRNA reads.
(PDF)

S6 Fig Characterization of RNAseq datasets. A) Total library reads for each RNAseq library B) Principle component analysis of wild-type (n = 3 replicates) and *klp10A*^*RNAi*^ (n = 3 replicates) RNAseq libraries. C) Scatter plot showing mean genic abundance of *klp10A*^*RNAi*^ versus wild-type libraries.
(PDF)

S7 Fig Klp10A localization at the central spindle of GSCs/SGs. Localization of acetylated MTs (acMTs) (red), Klp10A (green), and DNA (blue) in the apical region of a wild type testis (A), and in a telophase GSC-GB pair of a wild type testis (B). Arrows point to central spindle. Bars: 5 μm.
(PDF)

S8 Fig Identification of cell cycle stage for analysis of Piwi-Vasa colocalization. A-C) Same images as Fig 4A–4C and 4D–4F) same images as Fig 4E–4G are shown with additional α-Tubulin (blue) and DAPI (gray) channels to precisely define their cell cycle stages. Cytoplasmic α-Tubulin staining (without MT bundles of central spindle MTs) combined with decondensed DAPI staining indicate cells in G2 phase (A, D). Spindle α-Tubulin staining and condensed chromosomes indicate metaphase (B, E). Remnant of central spindle (by α-Tubulin staining) and decondensed chromosome indicate G1 phase (or S phase) of the cell cycle (completion of telophase) (C, F).
(PDF)

S11 Fig Piwi stays in nuage after mitotic exit in *klp10A*^*RNAi*^ germ cells. A) GFP-Piwi is nuclear in interphase GSCs/SGs in control testes. B) GFP-Piwi colocalizes with Vasa at the nuage of interphase GSCs/SGs in *klp10A*^*RNAi*^ germ cells. Cytoplasmic Vasa and α-Tubulin staining as well as DAPI staining indicates that these cells are in interphase. GFP-Piwi (green), Vasa (magenta). Arrowhead points to nuage-localized Piwi in interphase *klp10A*^*RNAi*^ GSCs/SGs. Bars 5μm. C) Number of interphase GSCs/SGs with nuage-localized Piwi per testis. n = 30 testes per genotype. p value of t-tests is provided.
(PDF)
