# Supplementary figures and images for: A kinesin Klp10A mediates cell cycle-dependent shuttling of Piwi between nucleus and nuage
Source: PLoS Genet. 2020 Mar 13;16(3):e1008648. doi: 10.1371/journal.pgen.1008648 (PMC7094869; doi:10.1371/journal.pgen.1008648)

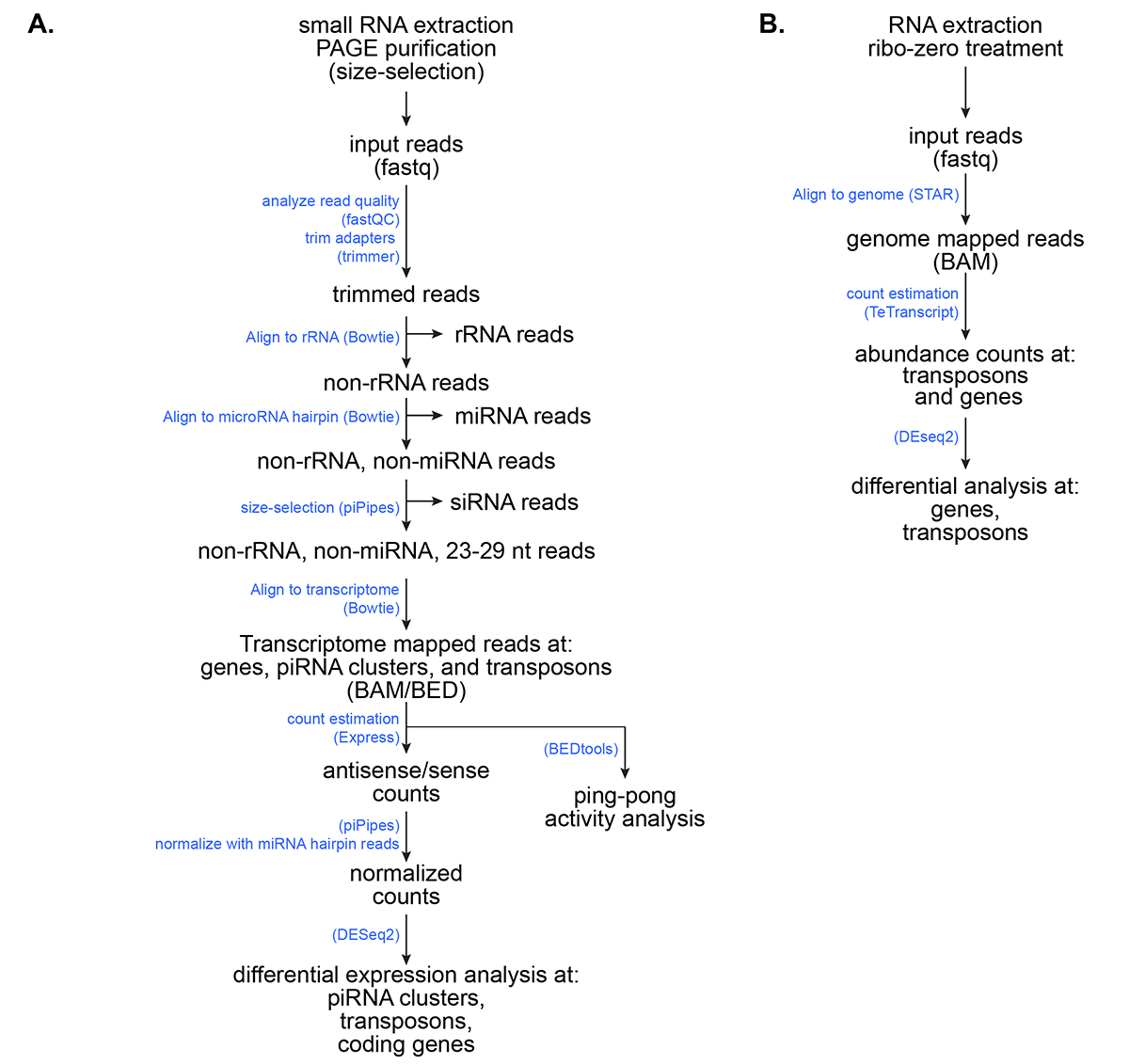

Supplement: S1 Fig — A) Pipeline of small RNA sequence analysis. B) Pipeline of mRNA sequence analysis. (TIF) [file pgen.1008648.s001.tif]

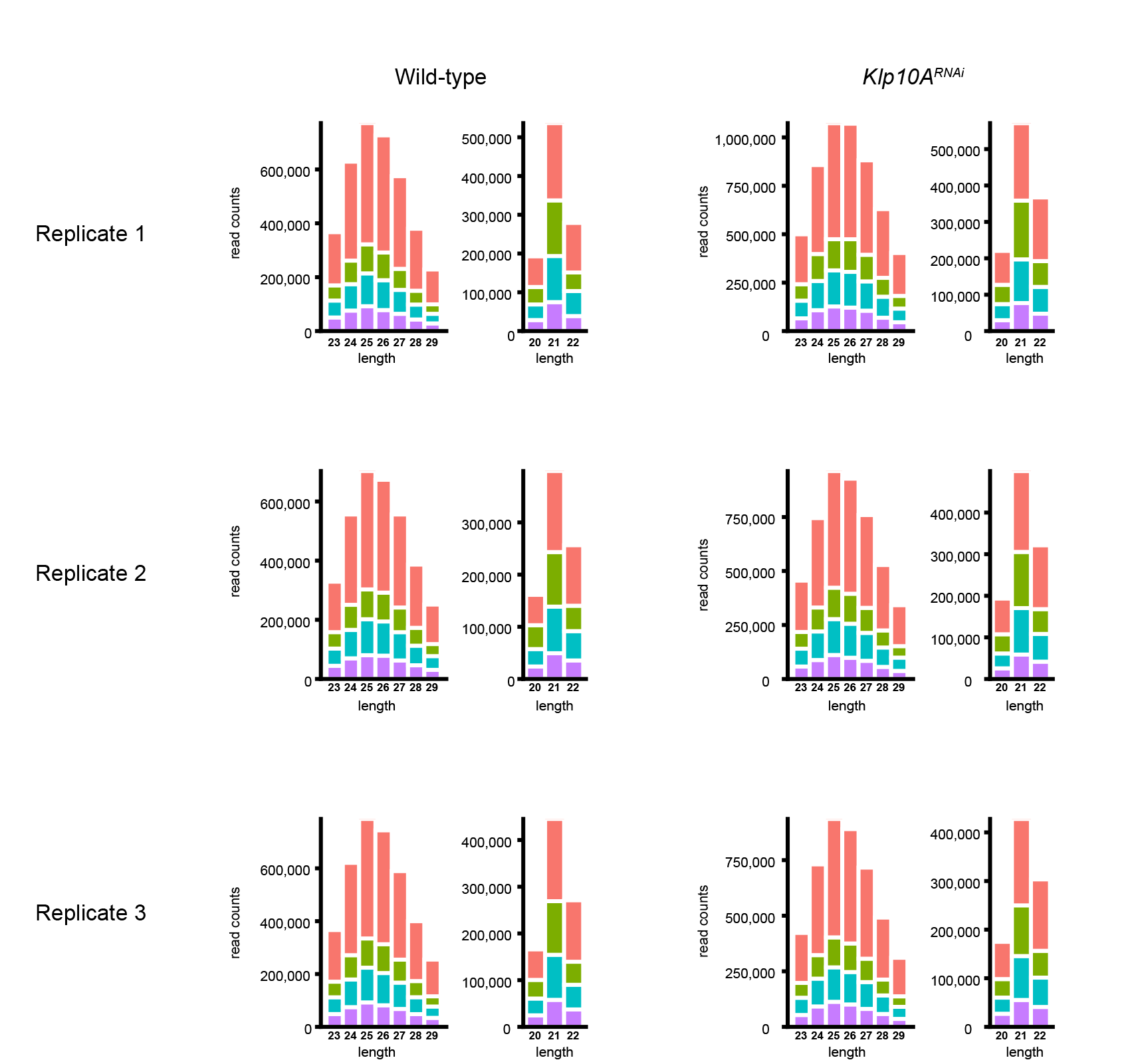

Supplement: S2 Fig — (TIF) [file pgen.1008648.s002.tif]

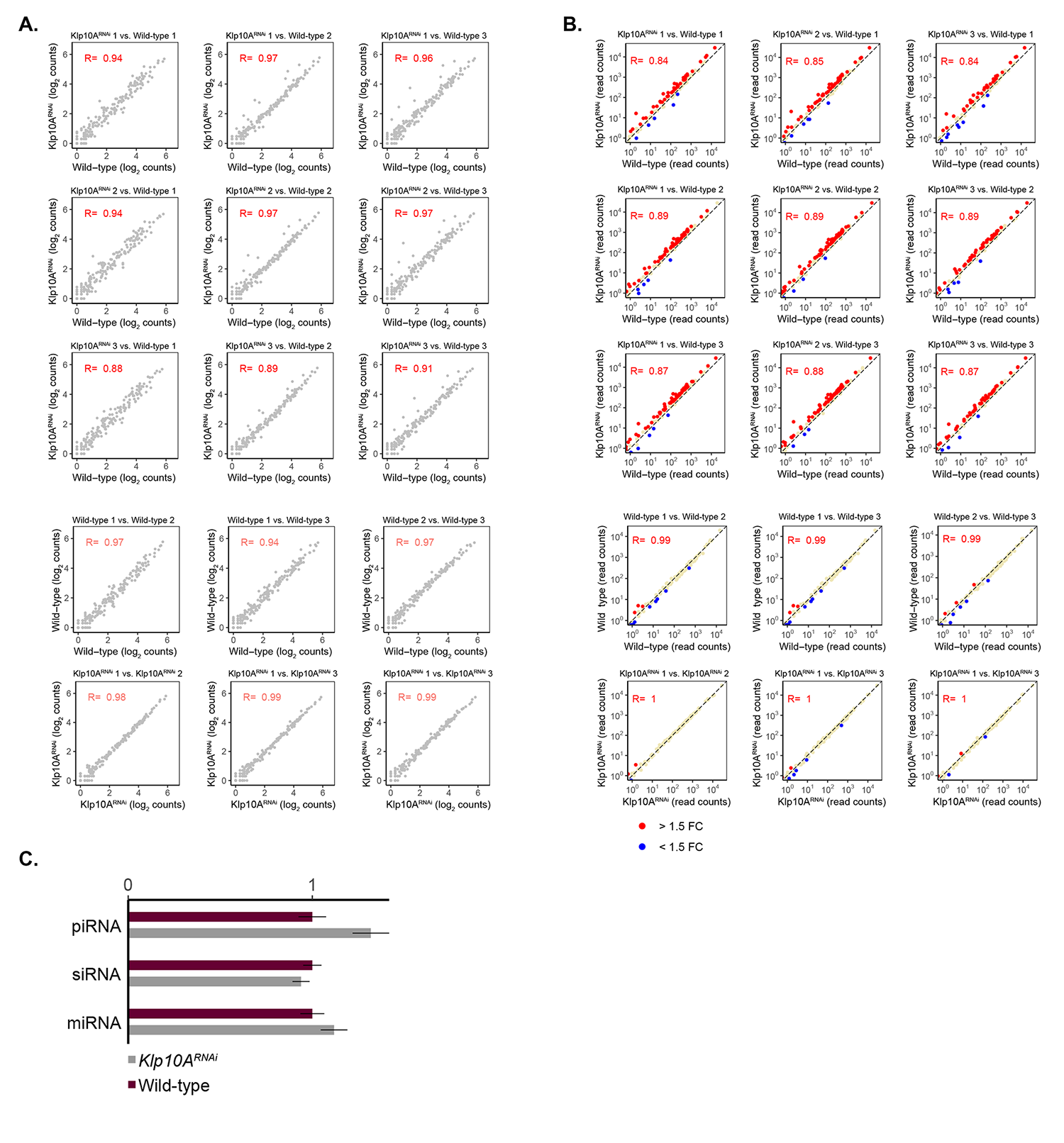

Supplement: S3 Fig — A-B) Pairwise comparisons of global miRNA hairpin abundance (A) and piRNA expression (B) between biological replicates. (C) difference in reads in different small RNA classes. Reads were normalized by total library reads. piRNA reads (23–29 nt) are increased in klp10ARNAi vs. wild-type, whereas siRNA (20–22 nt) or miRNA (reads mapping to miRNA hairpin features) reads are not. (TIF) [file pgen.1008648.s003.tif]

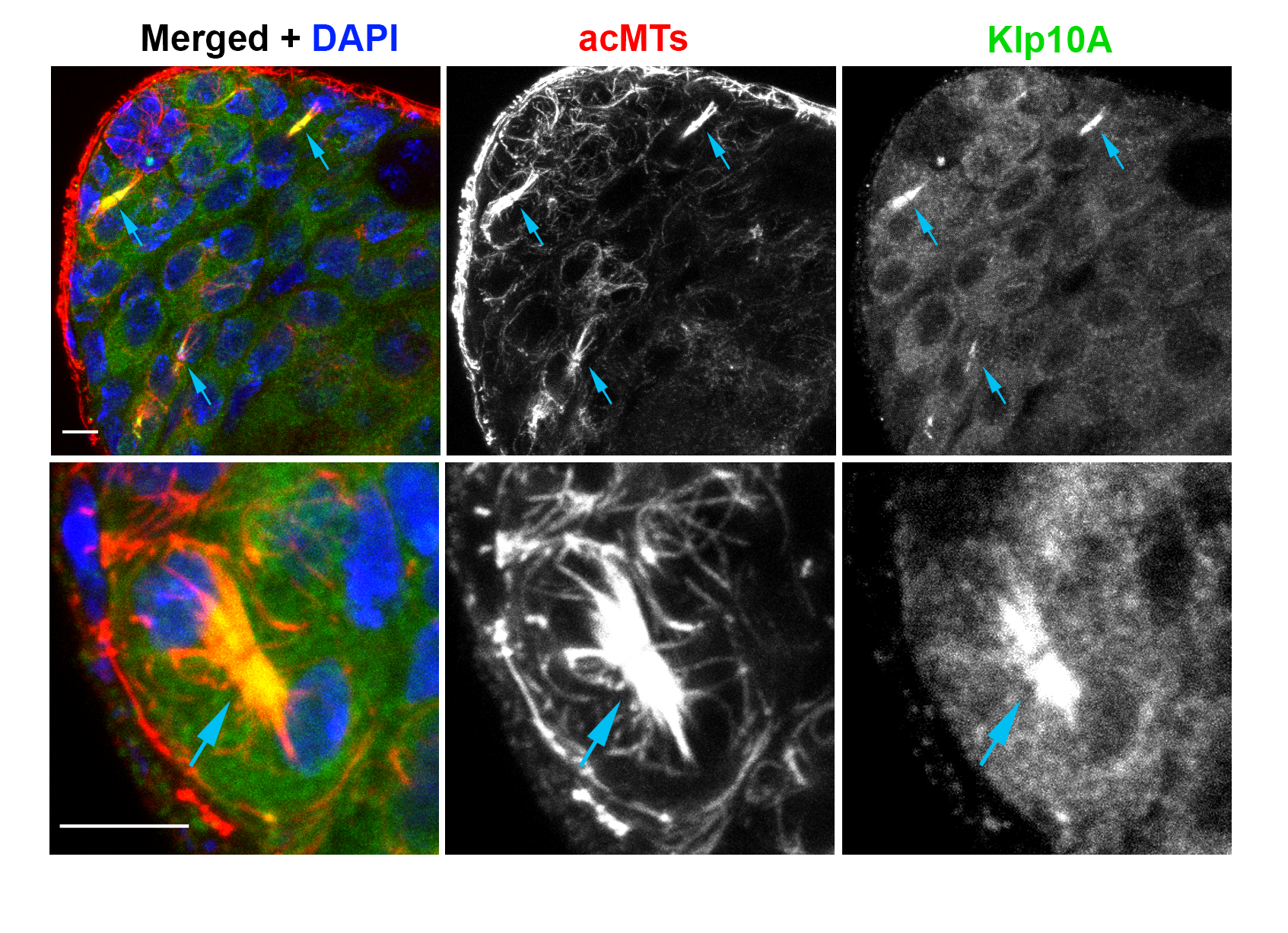

Supplement: S7 Fig — Localization of acetylated MTs (acMTs) (red), Klp10A (green), and DNA (blue) in the apical region of a wild type testis (A), and in a telophase GSC-GB pair of a wild type testis (B). Arrows point to central spindle. Bars: 5 μm. (TIF) [file pgen.1008648.s007.tif]

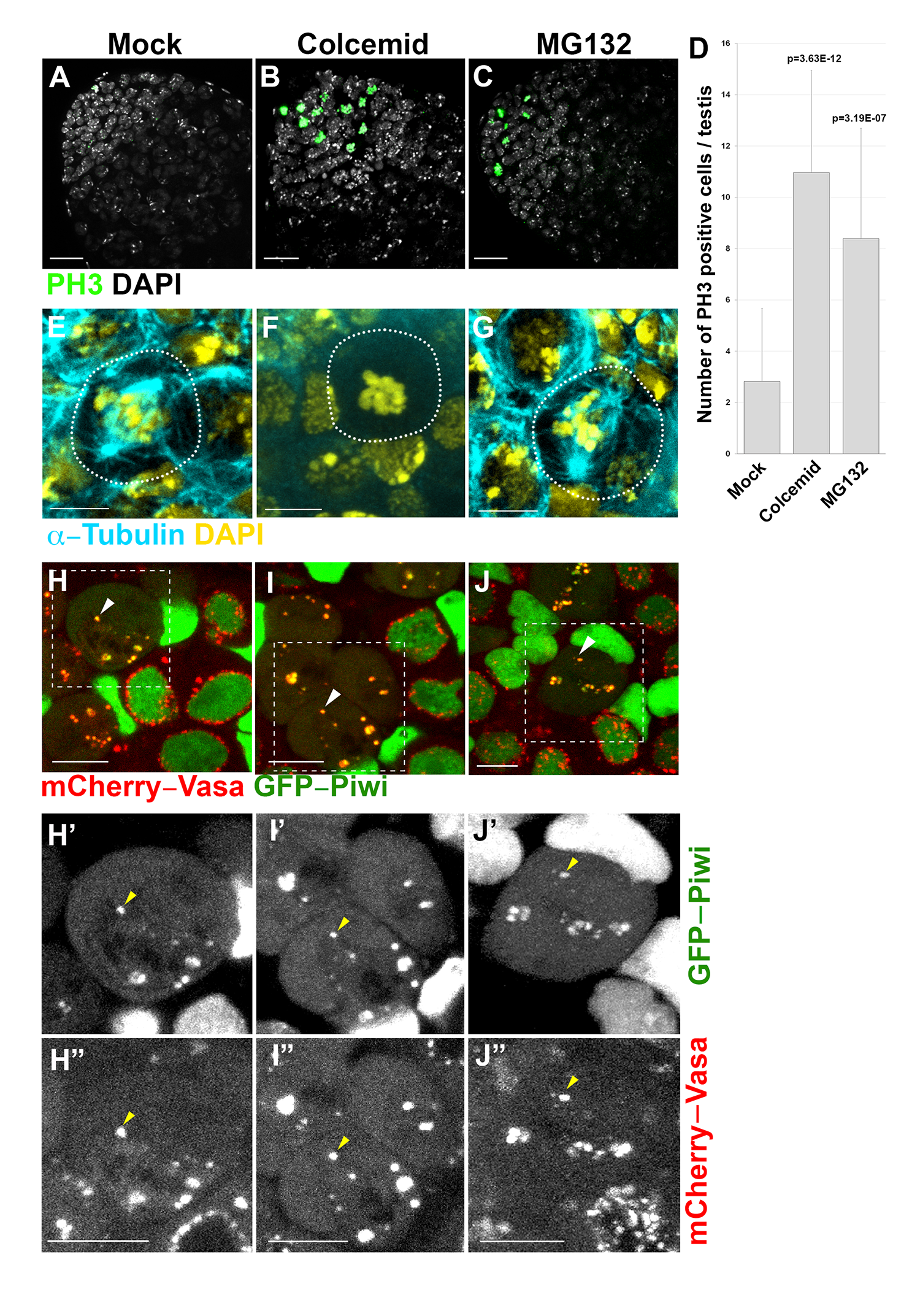

Supplement: S9 Fig — A-D) efficiency of mitotic arrest by colcemid or MG132. Apical tip of testes after 4.5h ex vivo mock (A), colcemid (B), or MG132 treatment (C). PH3 (green), DAPI (white). Bars: 20 μm. D) Number of mitotic cells per testis after 4.5h colcemid or MG132 treatment. Error bars indicate SD. P-values of t-tests are provided. E-G) Mitotic SGs after mock (E), colcemid (F), or MG132 (G) treatment. Colcemid efficiently depolymerizes MTs, whereas MG132 arrest cells in mitosis with intact spindle. α-Tubulin (cyan), DAPI (yellow). Bars: 5 μm. H-J) GFP-Piwi (green) and mCherry-Vasa (red) localization in SGs after 1h ex vivo culture with mock (H), colcemid (I) or MG132 (J) treatment. Magnified images of mitotic cells in H-I (boxed) are shown in H’-J”. Mitosis can be judged based on the lack of perinuclear Vasa localization and the lack of nuclear Piwi localization. Arrowheads point to mitotic nuage with Piwi-Vasa colocalization. Bars: 5 μm. (TIF) [file pgen.1008648.s009.tif]

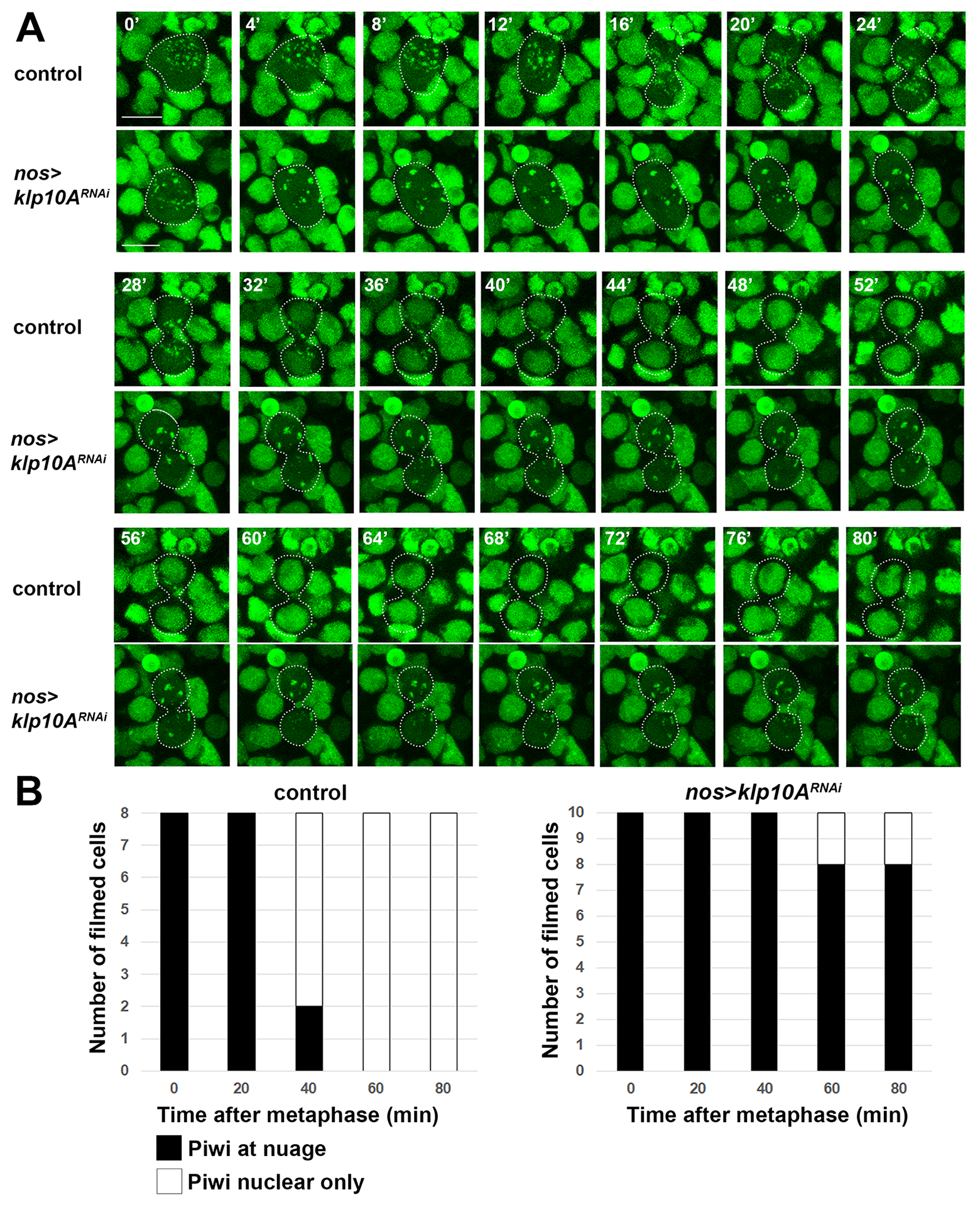

Supplement: S10 Fig — A) GFP-Piwi (green) localization during mitosis in a control or klp10ARNAi germ cells. Mitotic cells are encircled by dotted lines. Time in minutes. Bar: 5 μm. B) Quantification of GFP-Piwi localization during the mitotic exit of GSCs and SGs. (TIF) [file pgen.1008648.s010.tif]

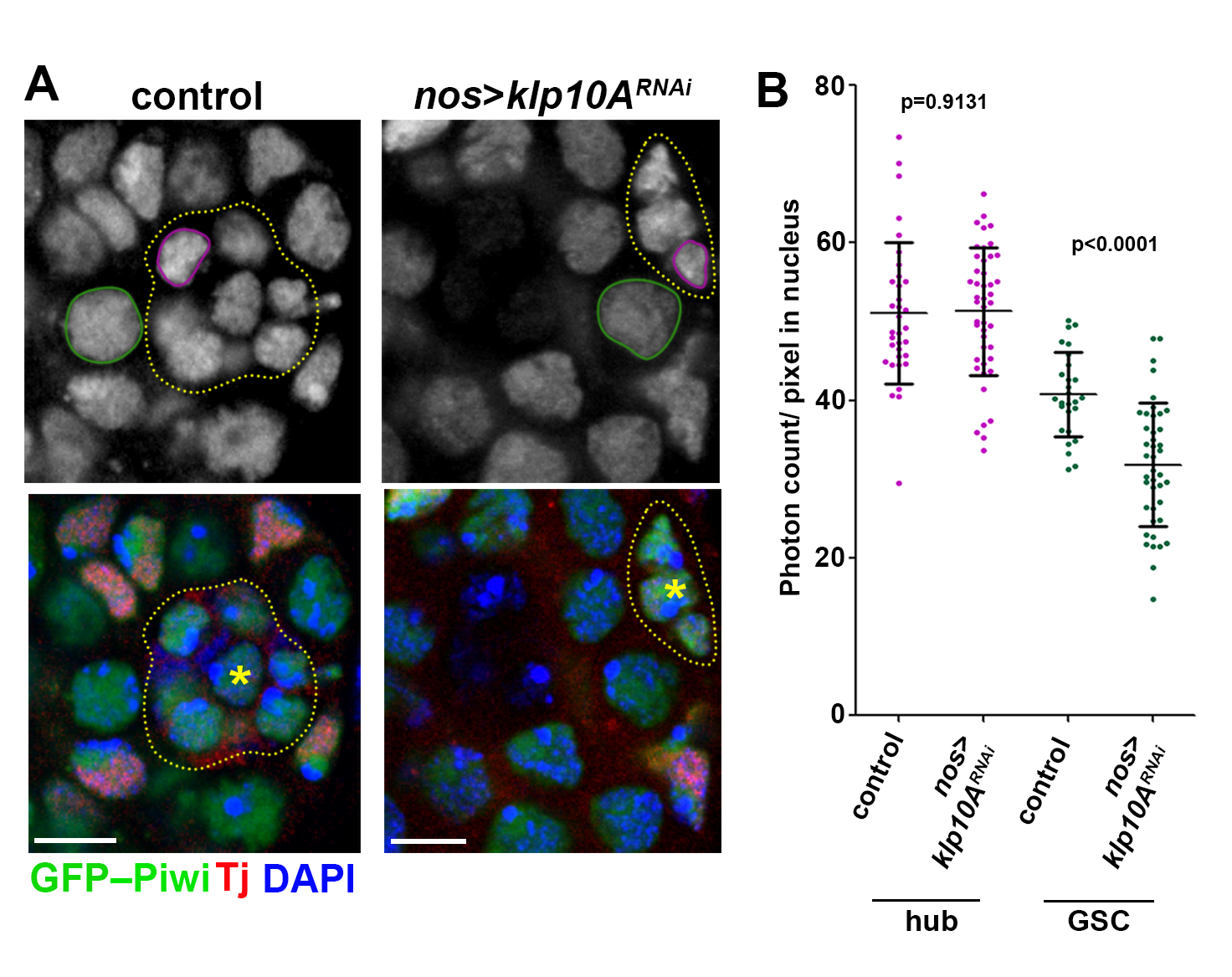

Supplement: S12 Fig — A) GFP-Piwi (green) in the apical tip of control and klp10ARNAi testes. Tj (red) identifies cyst stem cells and early cyst cells. DAPI (blue). The area surrounded by dotted lines with asterisks indicates hub. GSC nuclei are encircled by green line, hub cell nuclei by magenta line. Bars: 5 μm. B) Photon counts in nuclear areas of hub cells (magenta) and GSCs (green) in klp10ARNAi testes. Each data point represents individual single nucleus. Error bars indicate SD, p-values from t-tests are provided. As klp10A is knocked down only in germline (with nos-gal4), photon counts in hub cell nucleus is expected to be unchanged, and served as an internal control. Piwi nuclear level was reduced in GSCs upon knockdown of klp10A. Note that similar reduction in the nuclear Piwi was observed in SGs as well: however, hub cells and GSCs were used to quantify Piwi levels, because their juxtaposition allows accurate comparison. (TIF) [file pgen.1008648.s012.tif]

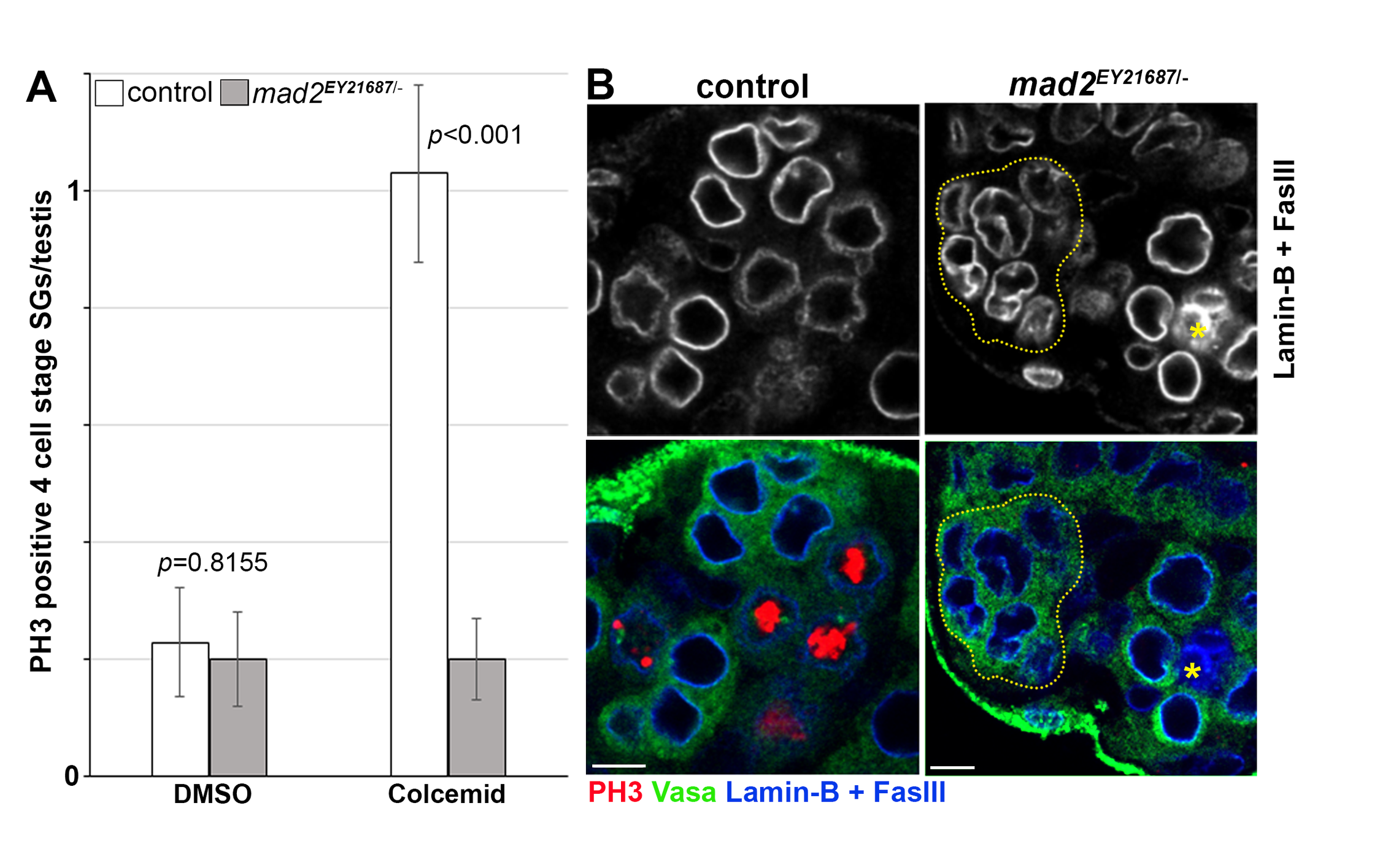

Supplement: S13 Fig — A) mad2 mutant SGs do not arrest in mitosis after clocemid-induced MT depolymerization. B) After prolonged MT depolymerization with colcemid, some mad2 mutant SGs exit mitosis: their nuclei are slightly larger because they are tetraploid, having exited mitosis without chromosome segregation (yellow dotted line indicates SGs that exited mitosis). Vasa (green), PH3 (red), Lamin-B and FasIII (blue). Bars: 5 μm. (TIF) [file pgen.1008648.s013.tif]
