# Supplementary material for: A kinesin Klp10A mediates cell cycle-dependent shuttling of Piwi between nucleus and nuage
Source: PLoS Genet. 2020 Mar 13;16(3):e1008648. doi: 10.1371/journal.pgen.1008648 (PMC7094869; doi:10.1371/journal.pgen.1008648)
Supplement: Attachment — Submitted filename: Klp10A-Piwi-response-Jan21-2020-fin.docx [file pgen.1008648.s024.docx]

Dear Gio,

Thank you very much for editing/reviewing our manuscript.

We very much appreciate the constructive comments provided by you and the reviewers.

We are confident that we have addressed all of the comments raised, as detailed below. After discussing plans for potential revisions with the authors, we concluded that we could respond to all of the comments thoroughly without the need for new experiments (new analysis of the data has been added). However, if you or the reviewers feel strongly that additional experiments are required, we would be willing to consider them; if so, we hope you will extend the deadline for submitting the official revision.

Thank you very much for your consideration, and we are looking forward to hearing from you.

Best wishes,

Yukiko

**Editor** comments (the part relevant to revision)

The reviewers appreciated the attention to an important topic but identified some aspects of the manuscript that should be improved. Although both reviewers were generally positive, both also raised a number of important points that need to be addressed.

We therefore ask you to modify the manuscript according to the review recommendations before we can consider your manuscript for acceptance. You may not need to add any additional or new data in order to address the reviewers' concerns, but if you choose to do so then reviewers will be asked to fully critique all new data. Please make sure that your revisions address all the specific points made by each reviewer. In particular, I would draw your attention to reviewer #1 comments regarding data presented in Figures 3&4.

We would like to thank the editor and reviewers for taking the time to review our manuscript and providing constructive comments. Below, we provide our point-by-point responses. We made particular efforts to address comments regarding Fig 3 and 4 thoroughly. We hope that the revised version is now acceptable for publication in PLOS Genetics.

Please note that this version of the manuscript contains Figure numbering for supplementary figures in the PLoS Genetics format, which will make it extremely difficult to track which figure is referred to in this response letter. Therefore, we have inserted new figure numbers in red: e.g. ‘Figure 2- supplementary Figure 1 (Supplementary Figure 1)’.

**Reviewer #1**: Piwi is an essential protein in metazoan germline maintenance and plays a major role in piRNA-mediated regulation of transposable elements. The mechanistic details of this important biology however remain unresolved. Understanding of Piwi dynamics during cell cycle adds to the mechanistic understanding and will contribute to the elucidation of the mechanism of Piwi function.

This paper describes the relocalization of Piwi from the nucleus to nuage during mitosis. The authors find that Piwi separates from nuage late in mitosis to form granules that appear to associate with the central spindle and migrate along these microtubules to segregate between the dividing cells. The microtubule depolymerising protein Klp10A is required for the latter parts of this process. During the late phase of mitosis it is also associated with the central spindle, and interacts (possibly indirectly) with several nuage components (Aub, Vasa), and somewhat with Piwi. Furthermore, depletion of Klp10A affects piRNA biogenesis, although without major effect on TE silencing.

The story has interesting bones, but there seem to be quite some loose ends and discrepancies that make it difficult to fully evaluate. The data clearly support the movement of Klp10A from centrosome to central spindle and back, as well as the movement of Piwi from nucleus to nuage and back during cell cycle. However the connection between the two, as well as the functional relevance remains more unclear.

First, we would like to clarify a point regarding Piwi localization. It is the entire population of Piwi-containing mitotic nuage that interacts with the central spindle (not just the fraction of Piwi that is budding off, as this reviewer noted).

Whereas we agree that we do not have a full mechanistic explanation/understanding of how Klp10A facilitates Piwi’s dissociation from mitotic nuage (which is fully addressed in the Discussion section), we believe that the following results described in this manuscript provide sufficient support for our claim in this paper regarding the connection between the changes in Piwi localization and Klp10A’s function.

1. Klp10A interacts with Piwi/Ago3/Aub (the nature of its interaction, which was pointed out by this reviewer, is further discussed below) (Fig 1).
2. Klp10A is required for Piwi’s dissociation from nuage (Fig 5A-D).
3. Microtubules are required for Piwi’s dissociation from nuage (Fig5 E-G).
4. Klp10A regulates central spindle microtubules (Fig 6).

Although we also agree with the reviewer that the mechanistic details of how Klp10A impacts the piRNA pathway is somewhat unclear at this point, piRNAs are clearly upregulated in klp10A-RNAi (Fig 2), which we consider a significant finding. As to why this does not lead to change in TEs is addressed in Discussion as follows:

“ The failure of *klp10A* knockdown to show depletion of a majority of TEs, despite elevated levels of cognate piRNAs, could be because *klp10A* influences piRNA biogenesis only during limited stages of spermatogenesis, i.e. GSCs and SGs. For example, TE transcripts are not regulated by the piRNA pathway in germ cells of certain stages of spermatogenesis (Vourekas et al., 2012), and defects caused by *klp10A* depletion may be masked by unchanged TE expression in other stages of spermatogenesis. “

In summary, we believe that our report provides a sufficient body of information to make a solid conclusion, instead of having 'too many loose ends'. We think that what was perceived as ‘loose ends’ by the reviewer are future questions that will eventually lead to a deeper mechanistic understanding, rather than inconsistencies in our claim. Based on the comments/questions by this reviewer (below), we have revised our manuscript to better explain some of our data and make our conclusions clearer. We hope these efforts will now assuage the reviewer’s concerns of our original manuscript.

The authors show a genetic interaction between the movement of Piwi and klp10A, as it is also clear that the return to the nucleus is delayed in a Klp10A- background. However, this background leads to obvious mitotic defects as the central spindle remains, and the final stages of mitosis look disrupted. It is therefore difficult to determine whether the Piwi phenotype is a direct result of the Klp10A loss or a more indirect effect.

In the section describing Fig 2, we had already provided the information that klp10A-RNAi does not cause any cell cycle defects. In the klp10A mutant, the remnant of the central spindle MT stays longer than the wild type, but this is not the ‘telophase spindle’ and thus cells are not abnormally arrested in telophase. Instead, Klp10A-RNAi cells enter the next S phase with essentially normal timing, and progress through the cell cycle normally. It is only depolymerization of the central spindle/midbody MT that is delayed (Fig 6). The section describing Fig 6 has the following statement:

Importantly, increased frequency of germ cells with the central spindle is not due to skewed composition of cell cycle stages in *klp10A^RNAi^* germ cells, because *klp10A^RNAi^* germ cells had comparable frequency of being in S phase (22.6±8.6% EdU^+^ in control, *n*=89 GSC vs. 24.6±15.6% EdU+ in *klp10A^RNAi^*, n=103 GSC, n.s.) or in M phase (0.22±0.03 mitotic GSCs/testis in control, n=160 vs. 0.194±0.01 mitotic GSCs/testis in *klp10A^RNAi^*, n=222, n.s.).

We hope that this clarification addresses this reviewer’s concern.

The Mass Spec data is interpreted to support a direct role, but in fact Klp10A binds hundreds of proteins making it hard to know whether the finding that Piwi is one of them is meaningful. Furthermore the Mass Spec recovers Piwi in one experiment, but not in another, suggesting that this is not a stable interaction - as would be expected form the cell biology, but makes the interpretation harder. In particular, as soon as some RNA-binding proteins are part of the complex, RNAs and other RNA-binding proteins are likely to associate as well to some degree. Therefore the IP experiments should be repeated in the presence of RNase to give relevance to this interaction.

First, we would like to note that any IP-mass spec analysis often yields hundreds of potential interacting proteins, and we do not believe Klp10A is particularly promiscuous in protein-protein interactions. As Table 1 shows, Piwi was always pulled down by Klp10A, and we believe this reviewer’s notion that “mass spec recovers Piwi in one experiment but not in another’ is incorrect.

Regarding the involvement of RNA in mediating protein-protein interaction, it is formally possible that RNA mediates Klp10A’s interaction with Piwi/Aug/Ago3. However, we do not necessarily think that RNase treatment will yield meaningful results that impact our interpretations in this study. This manuscript clearly provides evidence that Klp10A impacts Piwi’s localization. Therefore, even if Klp10A’s interaction with Piwi/Aub/Ago3 is mediated through RNA, it does not diminish the fact that Klp10A has a clear role in regulating Piwi’s localization. The main text now contains the following passage to address this point:

“Although it remains elusive whether Klp10A directly binds to these proteins or perhaps through an intermediary such as RNA, the fact that Klp10A co-immunoprecipitates multiple piRNA pathway components indicates that their interaction may be of biological relevance, regardless of whether the interaction is direct or not.” (After this description, we go on to show phenotypic relationship, i.e. klp10A-RNAi impacting Piwi localization.)

Although we feel otherwise, if the editor/reviewer strongly feels that testing RNA-dependence is utterly important and the decision on this manuscript depends on conducting co-IP experiments +/- RNase treatment, we would consider adding such experiments. If so, we hope that the editor provides us with additional time for us to perform this series of co-IP experiments.

A second loose end is that it is unclear what the functional relevance of the delayed relocalization of Piwi is. There is a mild effect on piRNA levels, although it is unclear how normalisation has been done to arrive at this conclusion. I didn't find any description of spikes in the sequencing data, and therefore expansion of one class of small RNA might actually be a reduction in other classes. This notwithstanding, the effect on transposon expression is sporadic and very mild.

While we agree with the reviewer that a spike-in control is one valid strategy for assessing technical differences across samples that may occur during sequencing, we nevertheless do not see large differences in other small RNA classes. For example, microRNA and siRNA reads do not show significant differences between treatments. The normalization methods are clearly stated in the methodology – all small RNA libraries were normalized to microRNA hairpins. We have shown in the Figure 2 – Supplement 2 (Supplementary Figure 3) that Klp10A-RNAi does not show significant effect on microRNA abundance. The following new panel (re-analysis of previously reported data) is now included in Figure 2 - Supplement 2 (Supplementary Figure 6) to address the differences in total reads between different small RNA classes.

Although modest, Klp10A’s effect on piRNA abundance is consistent and statistically significant, as reported in the manuscript. In contrast, Klp10A’s impact on TE is indeed sporadic. Regarding this point, we have an unpublished result that klp10A-RNAi is highly compromised in ‘adapting’ to TE invasion (using a hybrid dysgenesis paradigm), indicating that klp10A-mediated shuttling of Piwi between nucleus and nuage plays a critical role when cells need to adopt to a new set of TEs, which is consistent with our overall model. We considered incorporating this data as evidence for ‘relevance’. However, we are hesitant to do so at this point because adding such data would raise even more questions than it answers (e.g. What is the course of new TE invasion? Exactly how does Piwi transmit the information between nuage and the nucleus during adaptation to new TE invasion? and so on). Therefore, whereas this piece of data gives us confidence with the results we report in our manuscript, we feel that adding this part is beyond the scope of the current manuscript.

We also would like to note that a recent well-received paper from ZZ Zhang’s lab (in Figure S3 of their paper) noted a ~2.8-fold change in piRNA levels to be meaningful ([Moon et al., *Dev. Cell.*, 2019](https://doi.org/10.1016/j.devcel.2018.10.011)).

Overall, I think there are some interesting observations that are of interest to the wider community. Focussing the story on the relocalization of Piwi and reducing the emphasis on the interaction with Klp10A could make it more cohesive. This however would require a better characterisation of the functionality of the process.

We hope that the clarifications we provide will mitigate these concerns, as summarized above.

Specific comments:

Mass Spec and coIP data. (Fig1, Table1, Fig 4)
There is a quite remarkable discrepancy between Mass Spec data and Western: Western shows solid co-IP of Ago3 and very faint co-IP of Piwi. Mass Spec barely shows Ago3, but shows more solid Piwi.
Is there any explanation for this effect?

Mass spec and western blot results are not generally supposed to yield ‘consistent’ results, as this reviewer assumes. Also, reciprocal co-IPs are not expected to necessarily show the same ‘(apparent) strength’ of protein-protein interactions. In detecting the interaction of protein A and B, if A participates in many distinct complexes, whereas B’s main interacting partner is A, pulling down B will yield strong signal for A, but pulling down A will not yield a strong signal for B. Accordingly, we do not believe our mass spec data and reciprocal co-IP data should be considered as inconsistent.

From Mass Spec it appears that Klp10A binds hundreds of proteins. It still binds nuage as well, but this could be very indirect. Once you start pulling on RNA, you might get all kinds of RNA-associated proteins.
Is the interaction RNA-dependent?
The same goes for the coIP data in Fig 4:
The coIP data of Piwi and Vasa in Klp10A RNAi is intriguing, but this could again be just pulling on RNA and getting all kinds of other RNA-binding proteins in the same compartment.

As we discuss above, RNA-mediated interactions would still be meaningful. The defects in Piwi localization upon Klp10A-RNAi clearly suggest that the Klp10A-Piwi interaction (whether or not it is RNA dependent) is the basis for the changes in Piwi’s localization during the cell cycle.

Microscopy data. (Fig 3-6 and videos)
The microscope images are interesting, but it is hard to judge from them how representative the described relocalizations are, and how tightly temporally controlled the relocalization is even in the controls. It is noticeable that in images that don't focus on a protein, the localisation appears less discrete (e.g. Fig 3 Sup1 Vasa at telophase is not clearly on central spindle.)

We apologize for the confusion. We agree that Fig 3 sup1 image is not of an ideal quality and could have misled the reviewer. This is because the fixation conditions to optimally stain Vasa, acMT or Klp10A individually are not the same, and we have to compromise the staining quality to be able to simultaneously visualize all three. However, the main point of piRNA components colocalizing with Klp10A during telophase is well conveyed in main Fig 3, using non-compromising fixation conditions. The only point to be conveyed in Fig3-sup1 was that klp10A’s localization during telophase is central spindle. Therefore, we modified Fig3-sup1 (Supplementary Figure 7) to focus on conveying colocalization of klp10A and central spindle MTs only.

There also appears to be discrepancy in the staging between Fig 3 and Fig 4: in Fig 3 the point is that Piwi is close to the central spindle at telophase, but in Fig 4 it is already nuclear at telophase and definitely not on the central spindle.

We apologize for this confusion (this point was raised by reviewer #2 as well). Indeed, Fig 4 should be G1 phase. The labeling of telophase/G1 in Fig 4 is purely because of our convention of being careful (this is because we know that G1 is extremely short in GSCs, as published in [Inaba et al, *eLife,* 2015](https://elifesciences.org/articles/04960)) and we started using the term of ‘telophase/G1’ to be on the safe side for most cases. However, in this particular manuscript, where we additionally used microtubule staining etc., we actually can differentiate telophase from G1. Therefore, we should have labeled the panel as ‘telophase’ in Fig 3, and ‘G1’ in Fig 4. We have now revised this labeling. Also, Figure 4-supplement 3 (Supplementary Figure 10, movie frames) reveals the quite stereotypical progression of Piwi localization changes during the cell cycle, making us confident about our accurate identification of the cell cycle stages. We hope this clarification addresses the reviewer’s concern.

Furthermore, the localisation of Piwi to nuage depends much on seeing the right plane. As no nuage is in focus in the control panel it is impossible to tell if Piwi would have been in it or not. It would require full stacks or all east a plane with nuage in it to judge the difference.

This is not the case. The figures Fig4, Fig4-S1 (Supplementary Figure 8), Fig4-S2 (Supplementary Figure 9), Fig4-S4 (Supplementary Figure 11), contain co-staining between Piwi and Vasa, and in all of these panels, Piwi perfectly co-localizes with the nuage during mitosis (except for when Piwi is just about to leave the nuage, shown in Fig5). In control interphase cells, Vasa staining may not seem as restricted to the nuage due to differences caused by cytoplasmic staining and the smaller size of the nuage during interphase (we described in the manuscript that the nuage is much larger during mitotic stages when compared to interphase). But nuage granules is distributed all around the nuclear envelope, and thus the seeming lack of nuage granules in control is not due to an issue with the focal plane. For example, Fig4-S4 (Supplementary Figure 11) panel A shows control Vasa staining with much smaller nuage granules around the nuclear envelope, to which Piwi does not colocalize.

The budding off of the PIwi granule is intriguing, but very difficult to see. From the videos it appears that the overall Choreography of nuage is very different in Klp10A as it stays more peripheral. In the control, nuage does go to the region of the central spindle, but the association with central spindle itself seems at least incomplete.

We acknowledge that unbundled MTs (periphery of central spindle) may be harder to see in the movie. Moreover, movies show that nuage’s MT association can be temporal. These issues may make it harder to appreciate the budding-off process of Piwi from nuage. However, our manuscript provides ample evidence using multiple methods (movies, fixed samples, different reagents to mark different cellular components and distinct nuage components), and the results clearly show that 1) in control, mitotic nuage associates with the central spindle, and Piwi tends to leave mitotic nuage when it is interacting with central spindle MTs (please note that some MTs are difficult to see at the end of the central spindle, where multiple MTs are not bundled). 2) in klp10A-RNAi, Piwi does not leave nuage at the end of mitosis.

Further, in Klp10A RNAi Piwi localisation seems disorganised in general. Levels are lower, staining is patchy in some, and is overexposed in other images. This makes it difficult to judge how specific the relocalization of Piwi is in Klp10A-. In Fig4 Sup3 the DAPI signal even looks a bit weird and patchy in Klp10A-, suggesting there may be very general missegregation happening. Similarly, Fig 4 Sup 3 images clearly show a delay in the return of Piwi from nuage to nucleus. But is there a defined nucleus already? What does the nuclear membrane look like in klp10A-?

We described Piwi localization in Klp10A as follows:

1. The most prominent effect is Piwi’s delay in returning to the nucleus at the end of mitosis
2. Likely as a result, Piwi’s nuclear level is somewhat reduced (by ~20%, Fig4 sup5 [Supplementary Figure 12]).

Fig4 sup3 (Supplementary Figure 10) does not contain DAPI staining, and we are not sure what issues the reviewer is referring to. We also looked for other panels for ‘weird DAPI staining in klp10A’ in other Figures, but we could not find such figures (to our eyes). We have previously described that chromosome mis-segregation is quite infrequent in Klp10A-RNAi ([Chen et al., *eLife*, 2016](https://www.ncbi.nlm.nih.gov/pmc/articles/PMC5235350/)), and this work was cited in this manuscript in the context of explaining the lack of general mis-segregation in klp10A-RNAi. The concern that general mitotic delay is the cause of Piwi’s delayed return to the nucleus can be addressed by other figures that complement Fig4-sup3 (Supplementary Figure 10); for example, Fig4-sup4 (Supplementary Figure 11) panel B, where Piwi is clearly in cytoplasmic nuage in interphase of klp10A-RNAi (nuclear envelope formation is clear because subset of Piwi is nicely nuclear). And we have not observed nuclear envelope defects in Klp10A-RNAi using lamin staining.

The reviewer’s concern about ‘disorganized/patchy Piwi localization’ might also have come from the fact that the images contain other cell types (somatic cyst cells and hub cells), which have a very different nuclear shape and Piwi levels compared to germline cells, which is the focus of our study. In particular, the signal intensity of Piwi in somatic cells is more prominent when tissues are unfixed (upon fixation, the detection of somatic cell Piwi decreases for some unknown reason). For these reasons, we always use ‘dotted lines’ to demarcate germline cells of interest for each panel. We hope that these explanations address the reviewer’s concern.

Fig 6 actually also suggests that general segregation is altered as the central spindle persists after exit from mitosis, and videos 3 and 4 either are not from comparable stages, or show such severe segregation defect that none of the processes are recognisable in the mutant background.

Together this makes it difficult to judge how specific the described defect is.

As we explained above, we detect no cell cycle defects in klp10A-RNAi. Only the depolymerization of the remnant of central spindle MT is delayed. Evidence that cell cycle progression in klp10A-RNAi is normal include 1) overall frequency of S phase or M phase cells in klp10A-RNAi is similar to that of control, as described above. Fig6 shows how often acMT containing cells are in S phase, which is different from control. This was used to make the point that it is only acMT depolymerization that is influenced in klp10A-RNAi and not general cell cycle progression. 2) klp10A-RNAi in testis has normal differentiation progression, which involves constant GSC division, 4 rounds of mitotic divisions as spermatogonia, and 2 rounds of meiotic divisions. If ‘segregation defects’ were severe in klp10A-RNAi conditions, the testis would not have maintained normal morphology. The overall normal cell cycle progression is also documented in our previous paper ([Chen et al., *eLife*, 2016](https://www.ncbi.nlm.nih.gov/pmc/articles/PMC5235350/)). Therefore, we are confident with our conclusion described in this section.

Functional data (Fig 2)
It appears from the data that the ping-pong pathway is more active when Piwi remains in nuage longer. However, it is unclear how normalization is done. Could also be that miRNAs/tRNAs/rRNAs are down?

We did not compare rRNA reads between replicates due to depleting rRNA species during sample preparation. The technical variation between rRNA depletion could give confounding results. Instead, microRNA hairpin reads were used to normalize between small RNA libraries using the piPipes Suite ([Han et al., Bioinformatics, 2015](https://www.ncbi.nlm.nih.gov/pubmed/25342065))**.** This was stated in the methods section. Because we do not have a spike-in control, our rationale was to use other endogenous small RNA populations that do not shown significant changes across samples as an internal control. Accordingly, we chose to normalize to microRNA hairpin reads as is common practice in the piRNA field ([Senti et al., *Genes & Dev.*, 2015](http://genesdev.cshlp.org/content/29/16/1747.full); [Hayashi et al., *Nature*, 2016](https://www.ncbi.nlm.nih.gov/pmc/articles/PMC5164936/))**.** Justifying this normalization strategy, microRNA hairpin reads remain relatively equal between wild-type and klp10A-RNAi samples. We now show that microRNAs show no global changes between replicates (Fig2 – Supp6, Supplementary Figure 6). In addition, total microRNAs and endogenous siRNA levels show no significant change upon klp10A-RNAi.

Only a few TEs are affected (and only mildly). Is there a correlation between the affected TEs and the hyperactive ping-pong piRNAs?

As discussed in the manuscript (Discussion), we did not see clear correlation between affected TE vs. ping-pong piRNAs.

Overall this data is still puzzling as it would require increased piRNA cluster transcription as well as increased TE transcription to get higher levels of both sense and antisense piRNAs. Also, the ping-pong pathway is thought to mostly be formed by Ago3 and Aub, so how its overactivity is related to mislocalisation of Piwi is unclear.

We acknowledge that we do not have a full understanding of how compromised Piwi localization leads to the observed effects on piRNA/TE. However, the most straightforward interpretation (with currently available information) is that in klp10A-RNAi, Piwi fails to return to the nucleus. Cytoplasmic retention of Piwi would leads to a failure in silencing of genes/TEs that are supposed to be transcriptionally repressed by Piwi in the nucleus. As a result, genes/TEs are transcriptionally upregulated (albeit mild), then exported to the cytoplasm, and subsequently cleaved by the intact (not ‘overactivated’) ping-pong cycle, leading to upregulation of piRNA levels. We now explain our results and models more clearly in the Discussion section.

**Reviewer #2:** Yamashita and colleagues identify that Klp10A, a microtubule-depolymerizing kinesin motor protein, is required for proper shuttling of Piwi between the nucleus and cytoplasm, in what are presumed to be nuage, during mitosis. In the absence of Klp10A, piRNA levels are elevated and a small subset of transposons is downregulated, although the reason for this is not revealed in this study. Overall the results are interesting and could have important implications for further studies exploring nuage and piRNA dynamics.

The manuscript seems to have already undergone multiple rounds of revisions and I did not identify any major issues. A general concern, that I suspect may have derailed it at eLife, is the lack of solid mechanistic insight and a rather fuzzy link between microtubules and nuage and the piRNA machinery. Nonetheless, the results will likely be of broad interest and provide a valuable framework for future studies.

We fully agree that we do not have the full mechanistic understanding of how Klp10A mediates Piwi’s dissociation from the nuage (although klp10A is clearly required for this process). As mentioned above (response to reviewer #1), our data suggest that klp10A-mediated shuttling of Piwi might help the nucleus to adapt when new TEs come in. In the paradigm of hybrid dysgenesis, klp10A-RNAi is much more compromised upon introduction of a new TE (p-element). Based on these data, we speculate the following: when cells are dealing with TEs that are already familiar to cells, there is no need to update the repertoire of piRNAs, thus we do not see a drastic effect in klp10A-RNAi. But when a new TE invades, cells might have to shift the repertoire of piRNAs, by replacing old piRNA with new ones. To do so, Piwi has to shuttle between nuage granules and the nucleus, facilitated by klp10A. In the absence of klp10A, the nucleus cannot get ‘updates’ (transmitted by Piwi, that has communicated with the nuage during mitosis). However, these ideas are still at an early stage of development, and we feel that adding these data would only create more loose ends. We hope that the reviewer/editor agrees that knowing that Piwi and nuage interact in a cell cycle dependent manner, which is mediated by Klp10A, presents sufficient progress in the field, especially given that not much cell biology has been done in the field of piRNAs.

Minor Points

Lines 132-133: "To enrich for GSCs, the self-renewal factor Unpaired (Upd) (Kiger et al., 2001; Tulina and Matunis, 2001) was expressed (nos-gal4>UAS-upd)." A bit more explanation would help here.

We have added the following description:

‘Upd is a ligand expressed by hub cells to activate the JAK-STAT pathway, which determines GSC self-renewal. It was shown that ectopic expression of Upd in germ cells (*nos-gal4>UAS-upd*) is sufficient to cause GSC-like tumors in the testis (Kiger et al., 2001; Tulina and Matunis, 2001).’

Lines 147-150: "Similar interactions between Klp10A and piRNA pathway components (Vasa, Piwi, Aub, Ago3) were observed when extracts from SG tumor was used (nos>dpp, (Kawase et al., 2004; Schulz et al., 2004; Shivdasani and Ingham, 2003))." The rationale for using SG tumor should be noted.

The text contains the following description:

“…suggesting that the Klp10A interaction with piRNA pathway components is not unique to GSCs.”

We didn’t originally have a ‘rationale’ to test Dpp. We started with the intention to identify GSC-specific centrosomal proteins as described in the main text. After pulling down piRNA pathway components, we realized that klp10A-piRNA pathway interaction is unlikely on the GSC centrosome. And their likely interaction on the central spindle appeared to be common in GSCs and SGs. Then we went back to do co-IP with SG extracts to confirm our suspicion.

Lines 166-167: "We defined piRNAs as reads of 23-29 nucleotides (nt) in length that did not map to microRNAs or ribosomal RNAs." What is the evidence that these criteria are sufficient to define piRNAs? Perhaps it would make more sense to focus specifically on the known piRNA clusters.

We are unsure what the reviewer asks as evidence that these criteria are sufficient. We filtered for RNAs that were 23-29 nucleotide, to remove other small RNAs like siRNAs that might hamper downstream analysis used for this paper. The distribution of piRNA length in *Drosophila* has been shown by many labs to range from 23-29 nt (e.g. [Aravin et al., *Dev. Cell.*, 2003](https://doi.org/10.1016/S1534-5807(03)00228-4); [Vagin et al., *Science*, 2006](https://science.sciencemag.org/content/313/5785/320.long); [Stein et al., *Nat. Comm.*, 2019](https://www.nature.com/articles/s41467-019-08803-z)).

Supplementary Figure 2 has been added to show the majority of reads used in this study are 23-29 nucleotides in length start with a Uridine at the 5’ most position which has shown to be a unique, conserved feature of mature piRNAs. Note that this is a re-analysis of the data that were already provided in our original manuscript. Reads that are 20-22 nucleotides in length, and what we define as siRNAs, do not show such enrichment. This evidence convinces us that the majority of reads used in our analysis represent mature piRNAs. Furthermore, the piRNA reads used in most of the downstream analysis are those that map to the piRNA cluster or transposons.

Figure 2A. Error bars are needed.

We have added standard error bars to the Fig 2A.

Figure 2D. Of the transposons that are downregulated, how many have elevated levels of piRNAs? I'm trying to get a sense for whether there is a trend in piRNA upregulation and transposon downregulation, aside from the Hobo and FB examples shown. This should be noted in the results.

This point was also brought up during the review process of our manuscript at *eLife*. There are about ~10 TEs that are significantly down-regulated shown in Fig 2, but these ~10 TEs did not seem to have any common characteristics/trends (such as upregulated piRNA, or a subclass of TEs they belong to etc). We have added this description to the main text as following:

“There were no clear common characteristics among these TEs that may explain as to why these TEs exhibits downregulation.”

Figure 2-Figure 2 supplement 2B. Only piRNA features upregulated or downregualted >1.5 fold are shown, whereas all miRNAs are shown, which makes comparing the two sets of plots rather uninformative, particularly since miRNAs are not color coded similarly to the piRNAs.

In both panels, all the small RNA (piRNA and miRNA) are shown as grey dots, but only in piRNA panel, upregulations/downregulations were highlighted with colors. As described above and in the main text, miRNA does not show significant up/down regulation in klp10A-RNAi.

Lines 217-218: "When cells enter mitosis, nuage became somewhat larger in size, which we refer to as 'mitotic nuage' hereafter (Figure 3C, G and K)." What defines these foci as nuage?

These foci are defined as nuage granules by the presence of Ago3, Aub, as well as Vasa: the major nuage components. By live observation (please see the figure below), one can clearly see that the interphase and mitotic nuage are continuous entity. In this movie frames, one can see that as soon as cells enter mitosis (4:45) Piwi changes its localization from being pan nuclear to nuage. By comparing this time point with one frame before (3:09), it is clear that nuage itself (visualized by Vasa+) did not change. The very same nuage granule grows in size gradually, which becomes obvious around 14:28. Although this movie frames provides unambiguous answer to this reviewer’s concern, we do not believe that this additional figure adds to the manuscript because all the information can be clearly inferred from other data within the manuscript. (If this data is deemed necessary, we will add to the main manuscript).

Lines 238-241. Why does the localization of Piwi during telophase differ so much between figures 3 and 4A?

As we described in our response to reviewer #1, this is entirely our fault of combining the labeling of telophase and G1 together. This had become the practice in our lab after we discovered that G1 is extremely short in GSCs ([Inaba et al., *eLife*, 2015](https://elifesciences.org/articles/04960)), and as soon as telophase is complete, cells are almost in S phase. Therefore, for most of our other papers, it was a conservative measure to call telophase/G1 as one population (when the distinction between these two phases was not important). However, in this specific paper, the distinction is rather clear, as we use microtubule staining, which can distinguish telophase spindle from the remnants of the central spindle/midbody. Based on this finer criterion, Fig3 is clearly telophase, and Fig 4 is clearly G1. Accordingly, we changed the labeling of these figures.

Piwi is just one of several piRNA factors identified as interactors of Klp10A, yet only Piwi localization is noted as being impacted by Klp10A knockdown. Can the authors speculate as to what Klp10A might be doing in relation to the other piRNA factors?

This manuscript’s main point is about how Piwi dissociates from the nuage at the end of mitosis to go back to the nucleus. Unlike Piwi, three major piRNA components Vasa, Aub and Ago3 stay in nuage through interphase and mitosis as described in this manuscript. Similarly, most of the known piRNA components (including Kumo, Qin, Tapas, Spn-E, Tudor, Tejas, Vreteno) are reported to localize to perinuclear nuage during interphase and not the nucleus. Therefore, we do not expect these proteins to ‘go back to nucleus’ at the end of mitosis (these proteins have never been reported to our knowledge to have nuclear localization in regards to the piRNA pathway function). Accordingly, despite a formal possibility that additional piRNA pathway components’ localization may be affected in Klp10A, we feel that this independent line of inquiry falls beyond the scope of the present study.

There are a few known ‘nuclear factors’ other than Piwi that are shown to be involved in the piRNA pathway. This includes Rhino, moonshiner etc., but these factors function in the transcription of the pre-piRNA cluster, a process that is not believed to be operating in male germline. Therefore, we did not think that this line of experiments is warranted.

**References:**

Aravin AA, Lagos-Quintana M, Yalcin A, Zavolan M, Marks D, Snyder B, et al. The Small RNA Profile during Drosophila melanogaster Development. Developmental Cell. 2003;5(2):337–50.

Chen C, Inaba M, Venkei ZG, Yamashita YM. Klp10A, a stem cell centrosome-enriched kinesin, balances asymmetries in Drosophila male germline stem cell division. eLife. 2016;5.

Han BW, Wang W, Zamore PD, Weng Z. piPipes: a set of pipelines for piRNA and transposon analysis via small RNA-seq, RNA-seq, degradome- and CAGE-seq, ChIP-seq and genomic DNA sequencing. Bioinformatics. 2014;31(4):593–5.

Hayashi R, Schnabl J, Handler D, Mohn F, Ameres SL, Brennecke J. Genetic and mechanistic diversity of piRNA 3′-end formation. Nature. 2016;539(7630):588–92.

Inaba M, Venkei ZG, Yamashita YM. The polarity protein Baz forms a platform for the centrosome orientation during asymmetric stem cell division in the Drosophila male germline. eLife. 2015;4.

Moon S, Cassani M, Lin YA, Wang L, Dou K, Zhang ZZ. A Robust Transposon-Endogenizing Response from Germline Stem Cells. Developmental Cell. 2018;47(5).

Senti K-A, Jurczak D, Sachidanandam R, Brennecke J. piRNA-guided slicing of transposon transcripts enforces their transcriptional silencing via specifying the nuclear piRNA repertoire. Genes & Development. 2015;29(16):1747–62.

Stein CB, Genzor P, Mitra S, Elchert AR, Ipsaro JJ, Benner L, et al. Decoding the 5′ nucleotide bias of PIWI-interacting RNAs. Nature Communications. 2019;10(1).

Vagin VV. A Distinct Small RNA Pathway Silences Selfish Genetic Elements in the Germline. Science. 2006;313(5785):320–4.
