# Supplementary material for: Correction: A kinesin Klp10A mediates cell cycle-dependent shuttling of Piwi between nucleus and nuage
Source: PLoS Genet. 2020 Oct 21;16(10):e1009147. doi: 10.1371/journal.pgen.1009147 (PMC7577429; doi:10.1371/journal.pgen.1009147)
Supplement: S4 Fig — A-B) Density of sequenced piRNAs (blue: sense; red: antisense) across FB-element and BARI1. (PDF) [file pgen.1009147.s001.pdf]

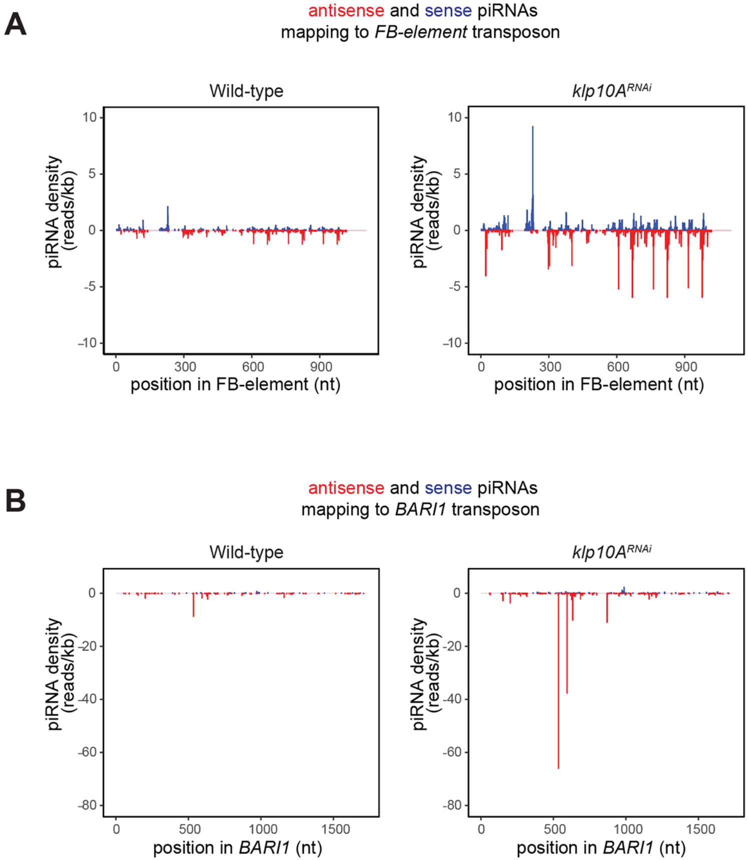
A
antisense and sense piRNAs
mapping to FB-element transposon
Wild-type
klp10A^RNAi
piRNA density (reads/kb)
10
5
0
-5
-10
0
300
600
900
position in FB-element (nt)
B
antisense and sense piRNAs
mapping to BARI1 transposon
Wild-type
klp10A^RNAi
piRNA density (reads/kb)
0
-20
-40
-60
-80
0
500
1000
1500
position in BARI1 (nt)
