# Supplementary figures and images for: Correction: A kinesin Klp10A mediates cell cycle-dependent shuttling of Piwi between nucleus and nuage
Source: PLoS Genet. 2020 Oct 21;16(10):e1009147. doi: 10.1371/journal.pgen.1009147 (PMC7577429; doi:10.1371/journal.pgen.1009147)

A.

Replicate 1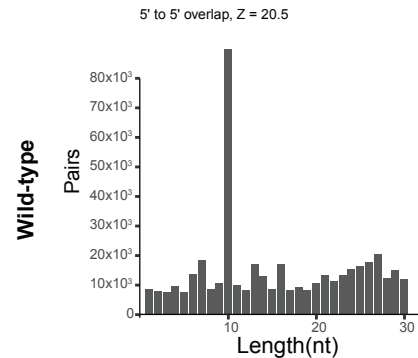Replicate 2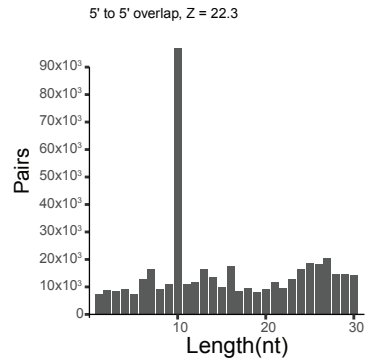Replicate 3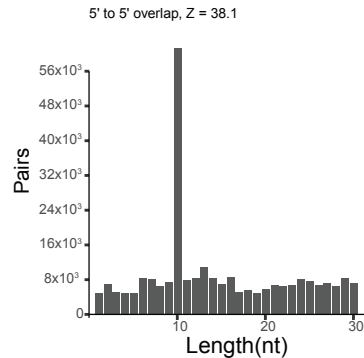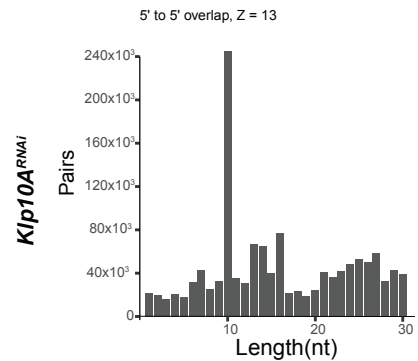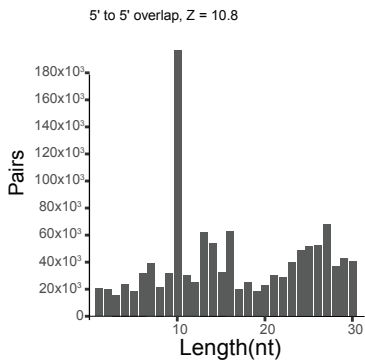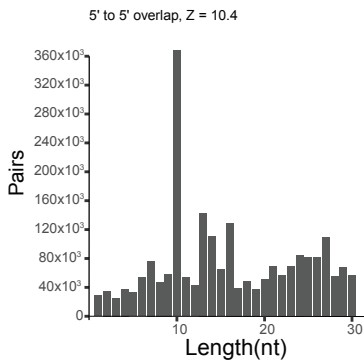

B.

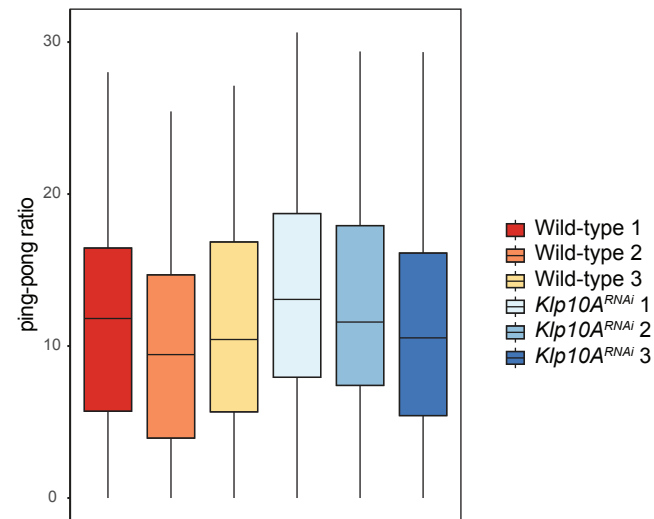

Supplement: S5 Fig — A) Histogram showing the distribution of antisense and sense piRNA pairs of piRNAs mapping to transposons. B) The box-plots show the distribution of ping-pong ratios of each transposon. Each box-plot is a different biological replicate. The Ping-pong ratio of each transposon was calculated by taking the sum of piRNA reads in which sense piRNAs with a 10 nt A and antisense piRNAs with a 1nt U showing 10 nucleotide complementarity from the 5’ end and dividing it with the total number of piRNA reads. (PDF) [file pgen.1009147.s002.pdf]

**A.**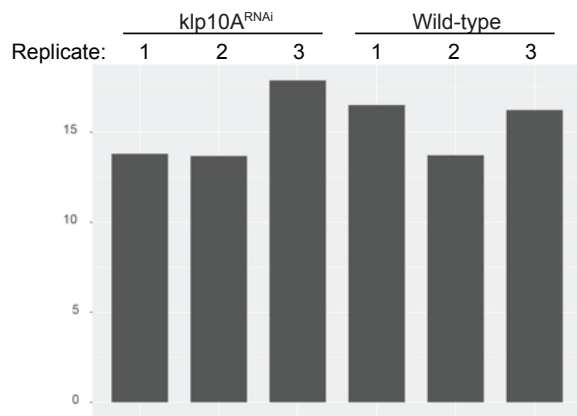**B.**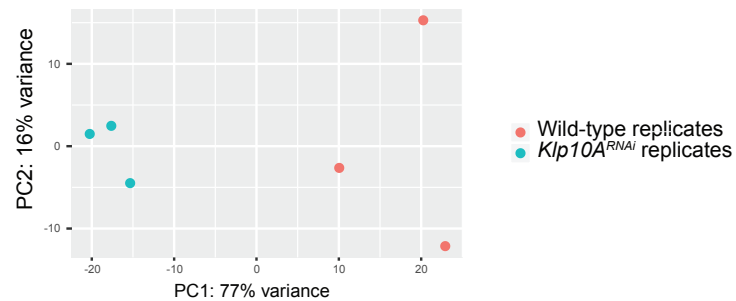**C.**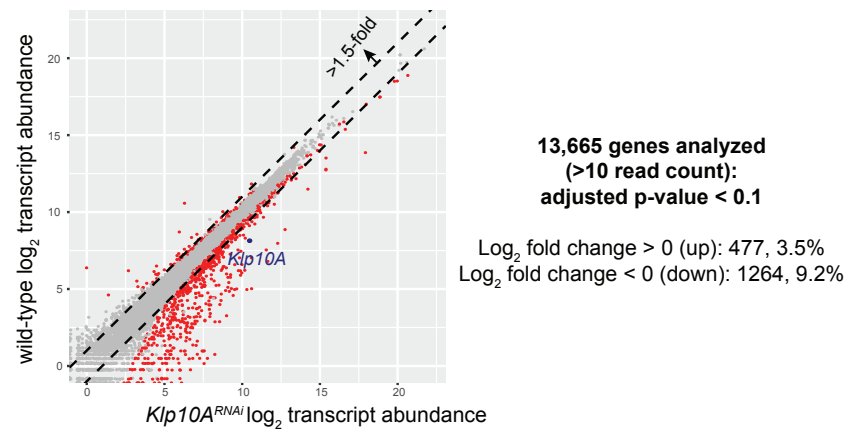

Supplement: S6 Fig — A) Total library reads for each RNAseq library B) Principle component analysis of wild-type (n = 3 replicates) and klp10ARNAi (n = 3 replicates) RNAseq libraries. C) Scatter plot showing mean genic abundance of klp10ARNAi versus wild-type libraries. (PDF) [file pgen.1009147.s003.pdf]

Merged + DAPI

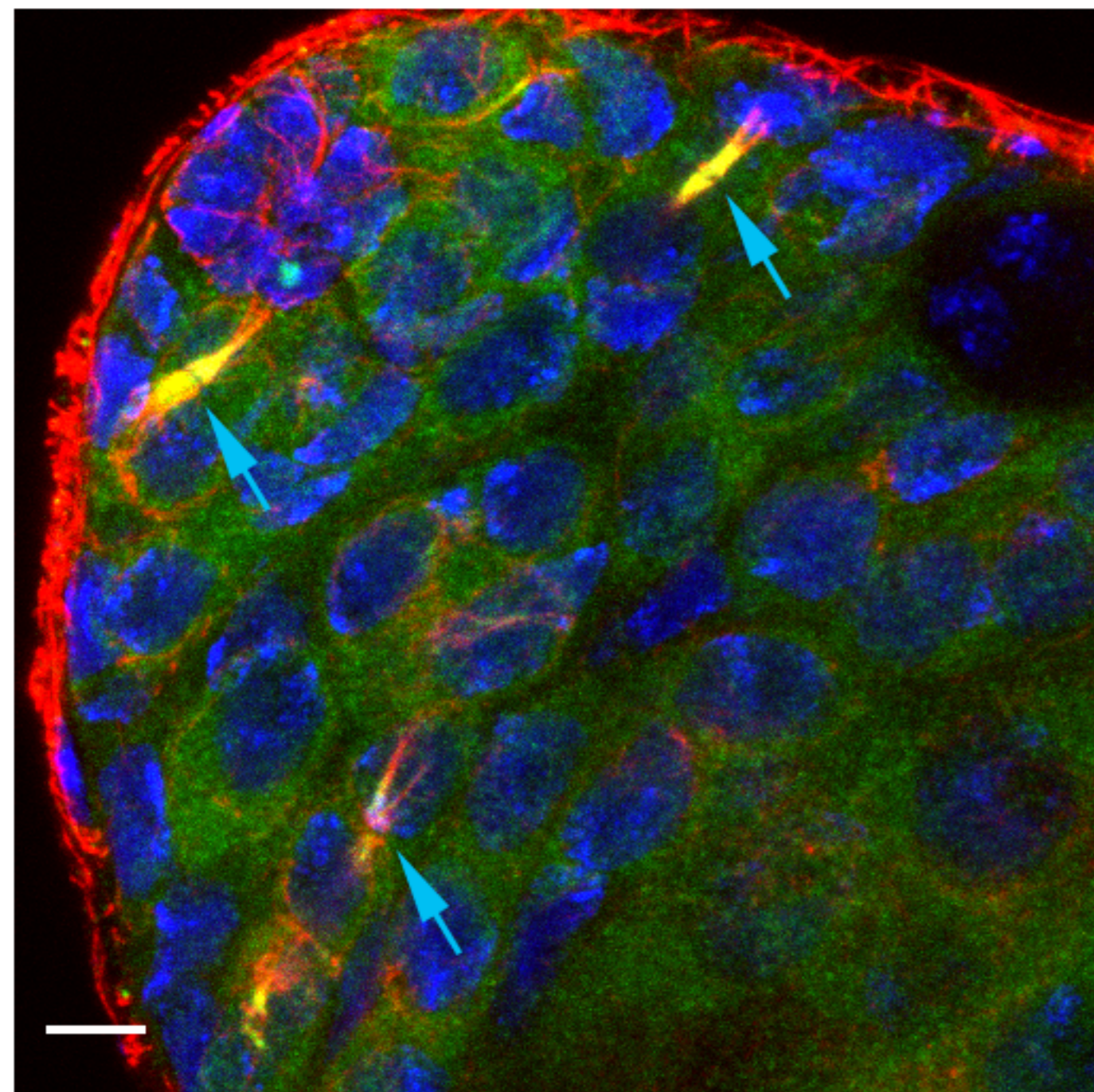

acMTs

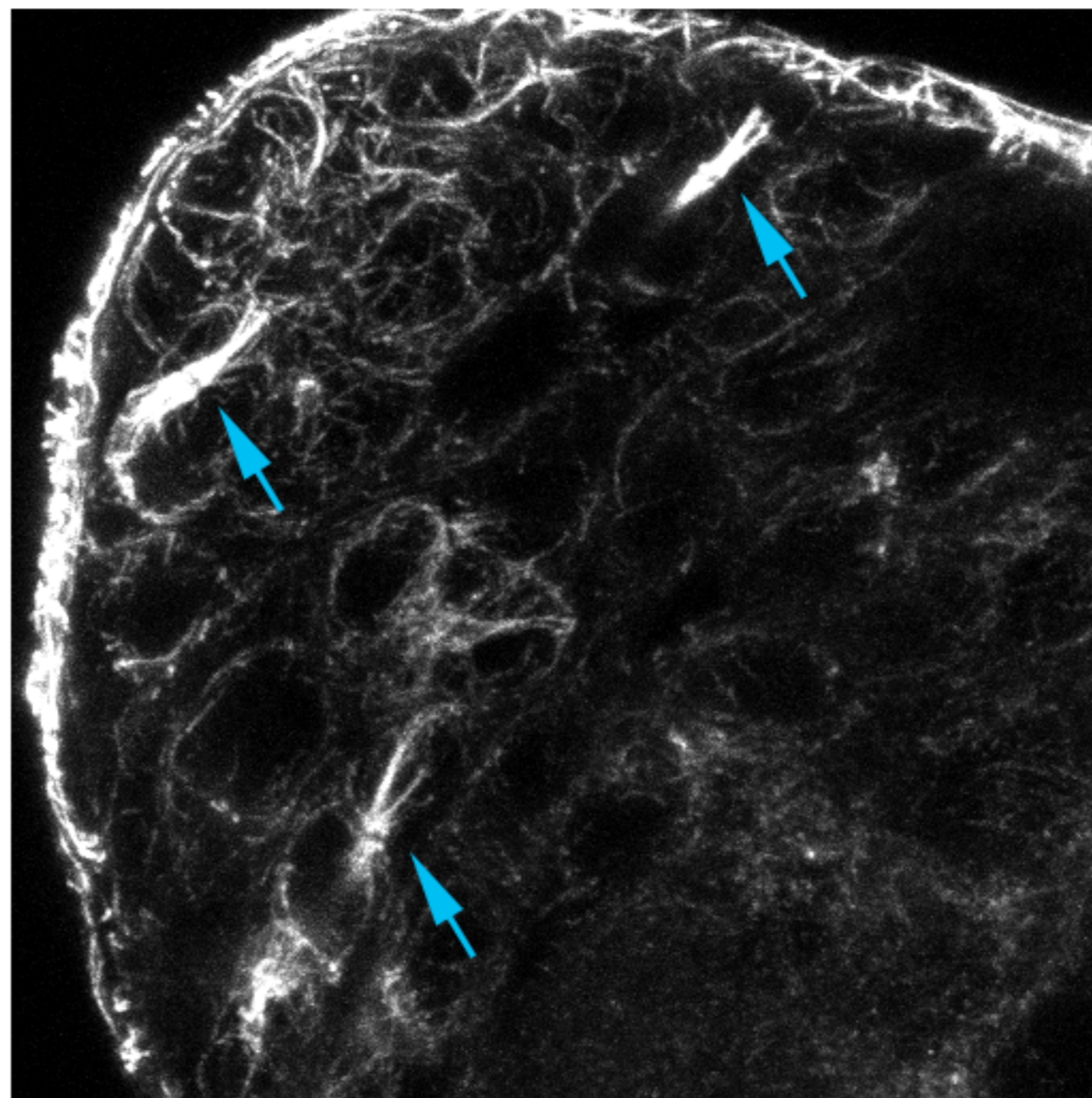

Klp10A

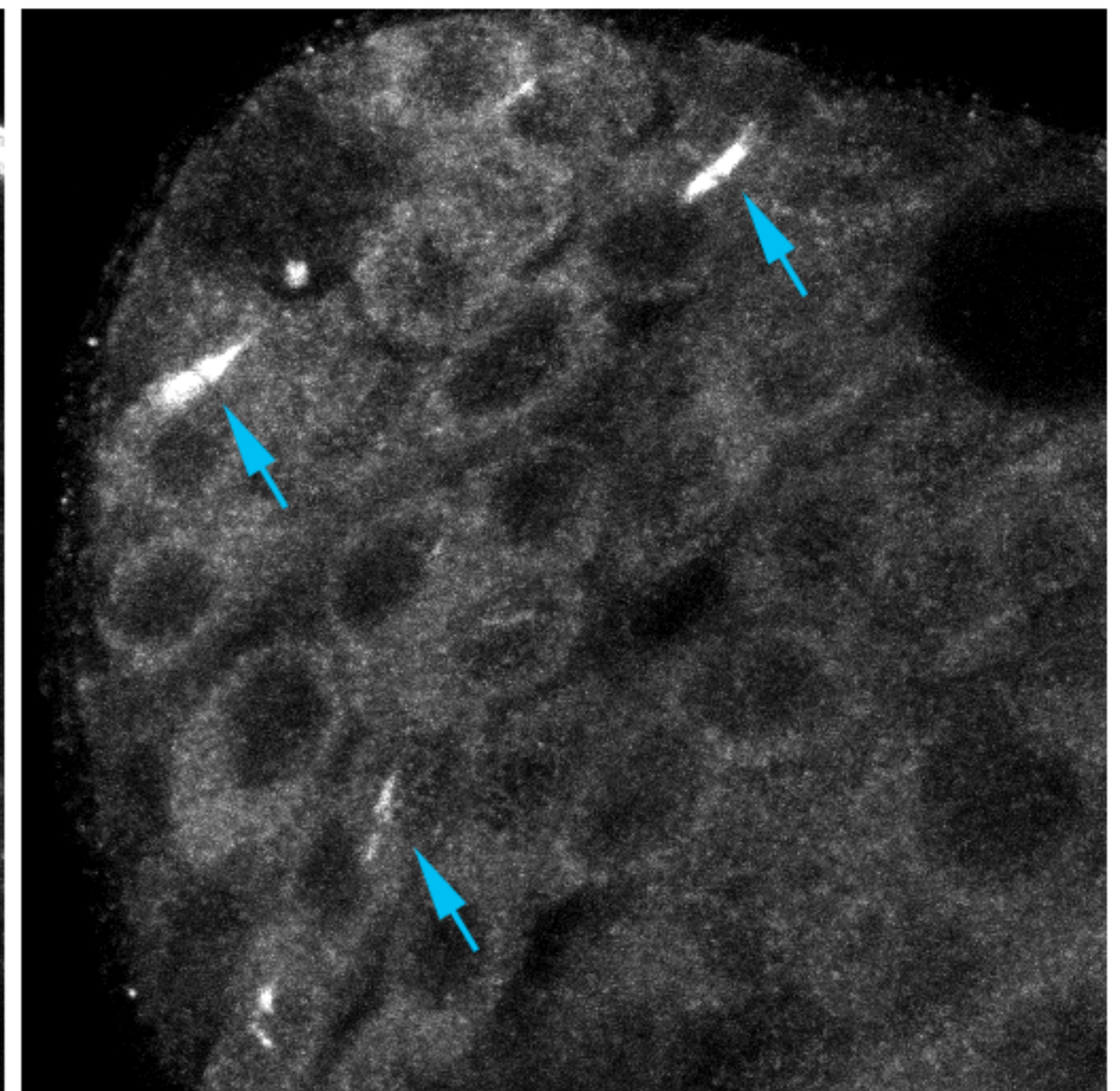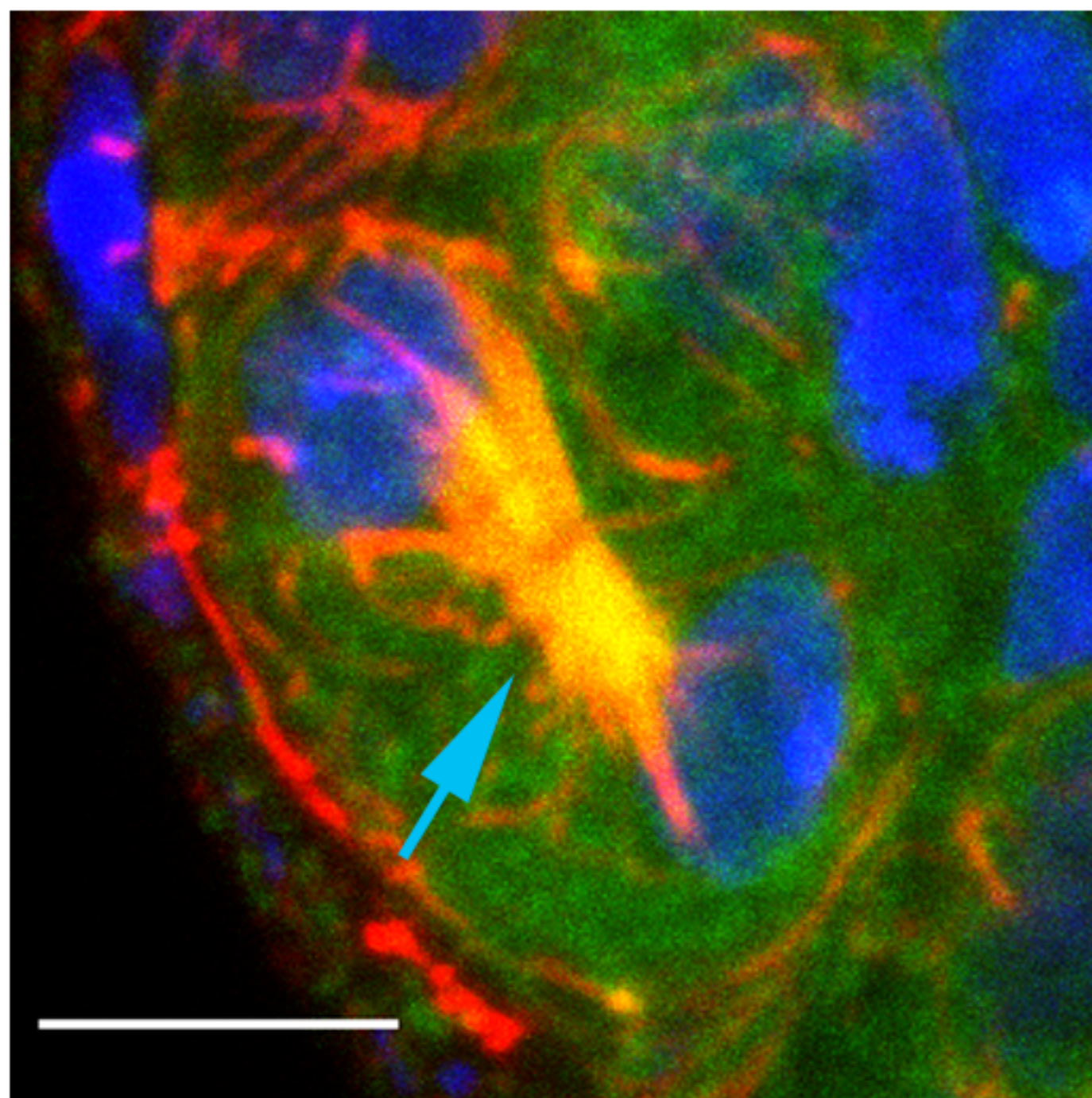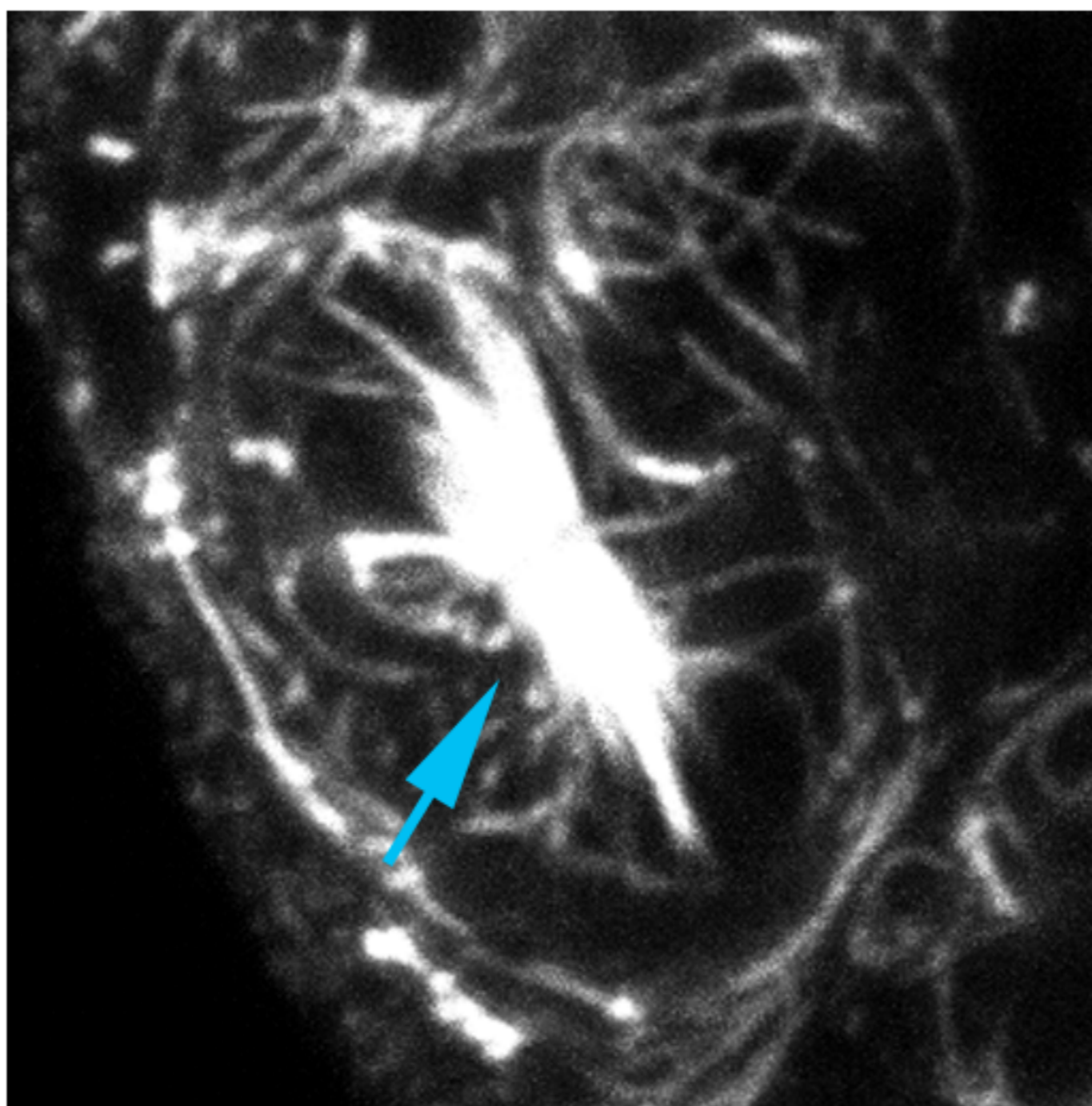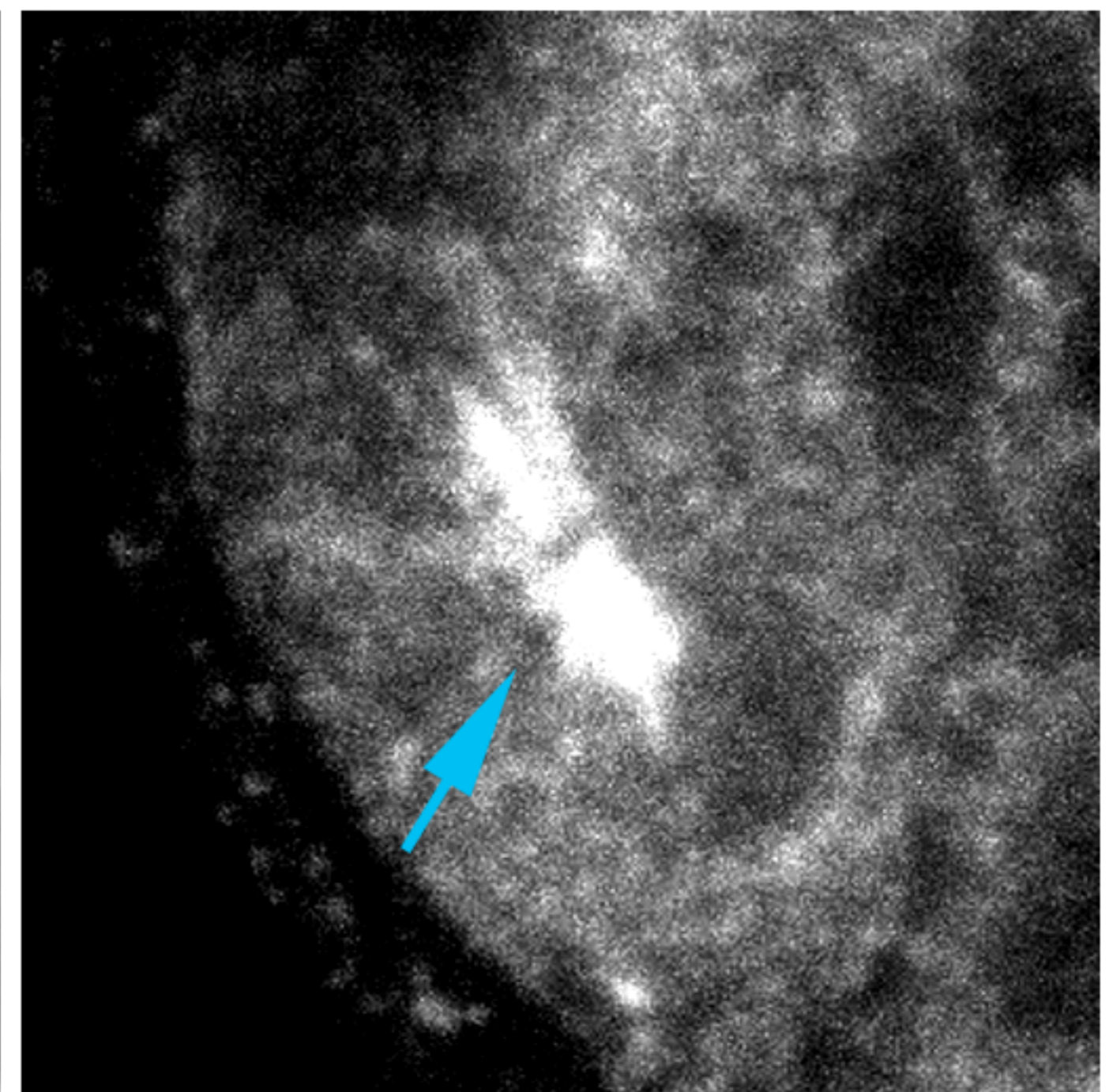

Supplement: S7 Fig — Localization of acetylated MTs (acMTs) (red), Klp10A (green), and DNA (blue) in the apical region of a wild type testis (A), and in a telophase GSC-GB pair of a wild type testis (B). Arrows point to central spindle. Bars: 5 μm. (PDF) [file pgen.1009147.s004.pdf]

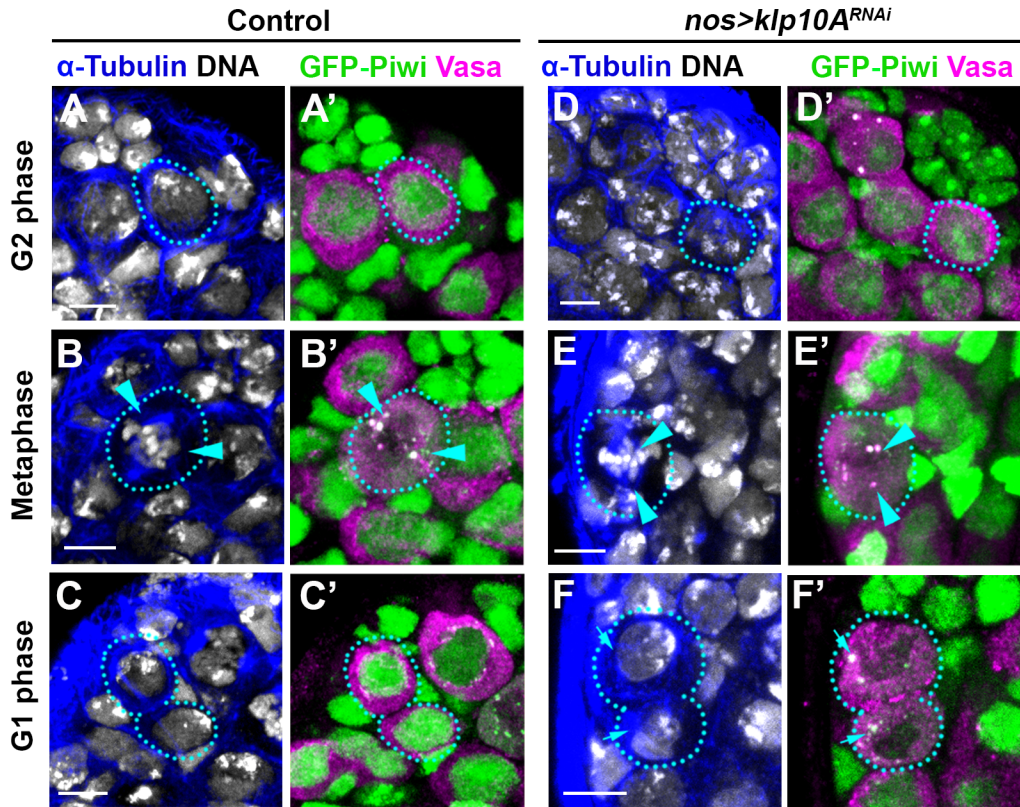

Supplement: S8 Fig — A-C) Same images as Fig 4A–4C and 4D–4F) same images as Fig 4E–4G are shown with additional α-Tubulin (blue) and DAPI (gray) channels to precisely define their cell cycle stages. Cytoplasmic α-Tubulin staining (without MT bundles of central spindle MTs) combined with decondensed DAPI staining indicate cells in G2 phase (A, D). Spindle α-Tubulin staining and condensed chromosomes indicate metaphase (B, E). Remnant of central spindle (by α-Tubulin staining) and decondensed chromosome indicate G1 phase (or S phase) of the cell cycle (completion of telophase) (C, F). (PDF) [file pgen.1009147.s005.pdf]
