# Supplementary material for: Correction: A kinesin Klp10A mediates cell cycle-dependent shuttling of Piwi between nucleus and nuage
Source: PLoS Genet. 2020 Oct 21;16(10):e1009147. doi: 10.1371/journal.pgen.1009147 (PMC7577429; doi:10.1371/journal.pgen.1009147)
Supplement: S11 Fig — A) GFP-Piwi is nuclear in interphase GSCs/SGs in control testes. B) GFP-Piwi colocalizes with Vasa at the nuage of interphase GSCs/SGs in klp10ARNAi germ cells. Cytoplasmic Vasa and α-Tubulin staining as well as DAPI staining indicates that these cells are in interphase. GFP-Piwi (green), Vasa (magenta). Arrowhead points to nuage-localized Piwi in interphase klp10ARNAi GSCs/SGs. Bars 5μm. C) Number of interphase GSCs/SGs with nuage-localized Piwi per testis. n = 30 testes per genotype. p value of t-tests is provided. (PDF) [file pgen.1009147.s006.pdf]

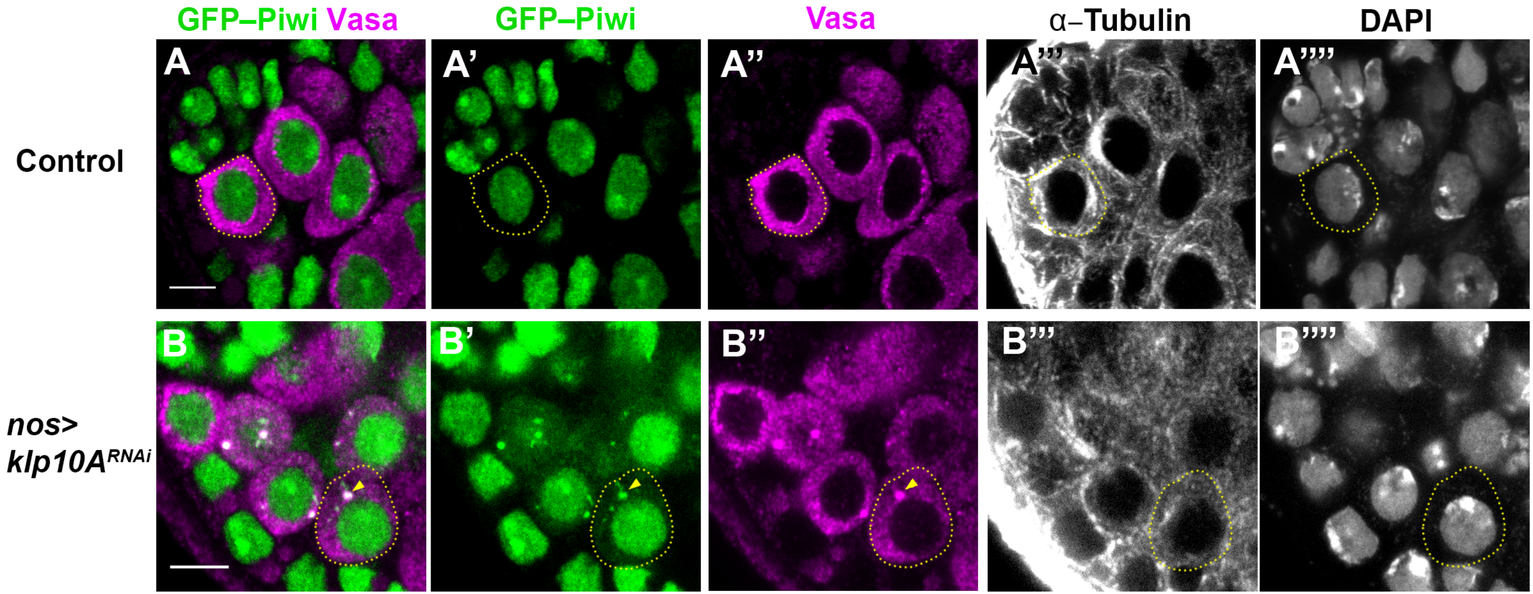

C

	GSC and SG			SC		
	control	*nos>klp10A*^{RNAi}	*p*-value	control	*nos>klp10A*^{RNAi}	*p*-value
**Interphase cells with cytoplasmic Piwi foci**	0.36±0.95	4.36±4.44	0.0001	0±0	0±0	n/a
